# Study of the production of charged pions, kaons, and protons in pPb collisions at $\sqrt{s_{NN}} =\;$5.02$\,\text {TeV}$

**DOI:** 10.1140/epjc/s10052-014-2847-x

**Published:** 2014-06-03

**Authors:** The CMS Collaboration, S. Chatrchyan, V. Khachatryan, A. M. Sirunyan, A. Tumasyan, W. Adam, T. Bergauer, M. Dragicevic, J. Erö, C. Fabjan, M. Friedl, R. Frühwirth, V. M. Ghete, N. Hörmann, J. Hrubec, M. Jeitler, W. Kiesenhofer, V. Knünz, M. Krammer, I. Krätschmer, D. Liko, I. Mikulec, D. Rabady, B. Rahbaran, C. Rohringer, H. Rohringer, R. Schöfbeck, J. Strauss, A. Taurok, W. Treberer-Treberspurg, W. Waltenberger, C.-E. Wulz, V. Mossolov, N. Shumeiko, J. Suarez Gonzalez, S. Alderweireldt, M. Bansal, S. Bansal, T. Cornelis, E. A. De Wolf, X. Janssen, A. Knutsson, S. Luyckx, L. Mucibello, S. Ochesanu, B. Roland, R. Rougny, Z. Staykova, H. Van Haevermaet, P. Van Mechelen, N. Van Remortel, A. Van Spilbeeck, F. Blekman, S. Blyweert, J. D’Hondt, A. Kalogeropoulos, J. Keaveney, M. Maes, A. Olbrechts, S. Tavernier, W. Van Doninck, P. Van Mulders, G. P. Van Onsem, I. Villella, C. Caillol, B. Clerbaux, G. De Lentdecker, L. Favart, A. P. R. Gay, T. Hreus, A. Léonard, P. E. Marage, A. Mohammadi, L. Perniè, T. Reis, T. Seva, L. Thomas, C. Van der Velde, P. Vanlaer, J. Wang, V. Adler, K. Beernaert, L. Benucci, A. Cimmino, S. Costantini, S. Dildick, G. Garcia, B. Klein, J. Lellouch, A. Marinov, J. Mccartin, A. A. Ocampo Rios, D. Ryckbosch, M. Sigamani, N. Strobbe, F. Thyssen, M. Tytgat, S. Walsh, E. Yazgan, N. Zaganidis, S. Basegmez, C. Beluffi, G. Bruno, R. Castello, A. Caudron, L. Ceard, C. Delaere, T. du Pree, D. Favart, L. Forthomme, A. Giammanco, J. Hollar, P. Jez, V. Lemaitre, J. Liao, O. Militaru, C. Nuttens, D. Pagano, A. Pin, K. Piotrzkowski, A. Popov, M. Selvaggi, J. M. Vizan Garcia, N. Beliy, T. Caebergs, E. Daubie, G. H. Hammad, G. A. Alves, M. Correa Martins Junior, T. Martins, M. E. Pol, M. H. G. Souza, W. L. Aldá Júnior, W. Carvalho, J. Chinellato, A. Custódio, E. M. Da Costa, D. De Jesus Damiao, C. De Oliveira Martins, S. Fonseca De Souza, H. Malbouisson, M. Malek, D. Matos Figueiredo, L. Mundim, H. Nogima, W. L. Prado Da Silva, A. Santoro, A. Sznajder, E. J. Tonelli Manganote, A. Vilela Pereira, F. A. Dias, T. R. Fernandez Perez Tomei, C. Lagana, S. F. Novaes, Sandra S. Padula, C. A. Bernardes, E. M. Gregores, P. G. Mercadante, V. Genchev, P. Iaydjiev, S. Piperov, M. Rodozov, G. Sultanov, M. Vutova, A. Dimitrov, R. Hadjiiska, V. Kozhuharov, L. Litov, B. Pavlov, P. Petkov, J. G. Bian, G. M. Chen, H. S. Chen, C. H. Jiang, D. Liang, S. Liang, X. Meng, J. Tao, X. Wang, Z. Wang, H. Xiao, M. Xu, C. Asawatangtrakuldee, Y. Ban, Y. Guo, W. Li, S. Liu, Y. Mao, S. J. Qian, H. Teng, D. Wang, L. Zhang, W. Zou, C. Avila, C. A. Carrillo Montoya, L. F. Chaparro Sierra, J. P. Gomez, B. Gomez Moreno, J. C. Sanabria, N. Godinovic, D. Lelas, R. Plestina, D. Polic, I. Puljak, Z. Antunovic, M. Kovac, V. Brigljevic, S. Duric, K. Kadija, J. Luetic, D. Mekterovic, S. Morovic, L. Tikvica, A. Attikis, G. Mavromanolakis, J. Mousa, C. Nicolaou, F. Ptochos, P. A. Razis, M. Finger, M. Finger, A. A. Abdelalim, Y. Assran, S. Elgammal, A. Ellithi Kamel, M. A. Mahmoud, A. Radi, M. Kadastik, M. Müntel, M. Murumaa, M. Raidal, L. Rebane, A. Tiko, P. Eerola, G. Fedi, M. Voutilainen, J. Härkönen, V. Karimäki, R. Kinnunen, M. J. Kortelainen, T. Lampén, K. Lassila-Perini, S. Lehti, T. Lindén, P. Luukka, T. Mäenpää, T. Peltola, E. Tuominen, J. Tuominiemi, E. Tuovinen, L. Wendland, T. Tuuva, M. Besancon, F. Couderc, M. Dejardin, D. Denegri, B. Fabbro, J. L. Faure, F. Ferri, S. Ganjour, A. Givernaud, P. Gras, G. Hamel de Monchenault, P. Jarry, E. Locci, J. Malcles, L. Millischer, A. Nayak, J. Rander, A. Rosowsky, M. Titov, S. Baffioni, F. Beaudette, L. Benhabib, M. Bluj, P. Busson, C. Charlot, N. Daci, T. Dahms, M. Dalchenko, L. Dobrzynski, A. Florent, R. Granier de Cassagnac, M. Haguenauer, P. Miné, C. Mironov, I. N. Naranjo, M. Nguyen, C. Ochando, P. Paganini, D. Sabes, R. Salerno, Y. Sirois, C. Veelken, A. Zabi, J.-L. Agram, J. Andrea, D. Bloch, J.-M. Brom, E. C. Chabert, C. Collard, E. Conte, F. Drouhin, J.-C. Fontaine, D. Gelé, U. Goerlach, C. Goetzmann, P. Juillot, A.-C. Le Bihan, P. Van Hove, S. Gadrat, S. Beauceron, N. Beaupere, G. Boudoul, S. Brochet, J. Chasserat, R. Chierici, D. Contardo, P. Depasse, H. El Mamouni, J. Fay, S. Gascon, M. Gouzevitch, B. Ille, T. Kurca, M. Lethuillier, L. Mirabito, S. Perries, L. Sgandurra, V. Sordini, M. Vander Donckt, P. Verdier, S. Viret, Z. Tsamalaidze, C. Autermann, S. Beranek, B. Calpas, M. Edelhoff, L. Feld, N. Heracleous, O. Hindrichs, K. Klein, A. Ostapchuk, A. Perieanu, F. Raupach, J. Sammet, S. Schael, D. Sprenger, H. Weber, B. Wittmer, V. Zhukov, M. Ata, J. Caudron, E. Dietz-Laursonn, D. Duchardt, M. Erdmann, R. Fischer, A. Güth, T. Hebbeker, C. Heidemann, K. Hoepfner, D. Klingebiel, P. Kreuzer, M. Merschmeyer, A. Meyer, M. Olschewski, K. Padeken, P. Papacz, H. Pieta, H. Reithler, S. A. Schmitz, L. Sonnenschein, J. Steggemann, D. Teyssier, S. Thüer, M. Weber, V. Cherepanov, Y. Erdogan, G. Flügge, H. Geenen, M. Geisler, W. Haj Ahmad, F. Hoehle, B. Kargoll, T. Kress, Y. Kuessel, J. Lingemann, A. Nowack, I. M. Nugent, L. Perchalla, O. Pooth, A. Stahl, M. Aldaya Martin, I. Asin, N. Bartosik, J. Behr, W. Behrenhoff, U. Behrens, M. Bergholz, A. Bethani, K. Borras, A. Burgmeier, A. Cakir, L. Calligaris, A. Campbell, S. Choudhury, F. Costanza, C. Diez Pardos, S. Dooling, T. Dorland, G. Eckerlin, D. Eckstein, G. Flucke, A. Geiser, I. Glushkov, P. Gunnellini, S. Habib, J. Hauk, G. Hellwig, D. Horton, H. Jung, M. Kasemann, P. Katsas, C. Kleinwort, H. Kluge, M. Krämer, D. Krücker, E. Kuznetsova, W. Lange, J. Leonard, K. Lipka, W. Lohmann, B. Lutz, R. Mankel, I. Marfin, I.-A. Melzer-Pellmann, A. B. Meyer, J. Mnich, A. Mussgiller, S. Naumann-Emme, O. Novgorodova, F. Nowak, J. Olzem, H. Perrey, A. Petrukhin, D. Pitzl, R. Placakyte, A. Raspereza, P. M. Ribeiro Cipriano, C. Riedl, E. Ron, M. Ö. Sahin, J. Salfeld-Nebgen, R. Schmidt, T. Schoerner-Sadenius, N. Sen, M. Stein, R. Walsh, C. Wissing, V. Blobel, H. Enderle, J. Erfle, E. Garutti, U. Gebbert, M. Görner, M. Gosselink, J. Haller, K. Heine, R. S. Höing, G. Kaussen, H. Kirschenmann, R. Klanner, R. Kogler, J. Lange, I. Marchesini, T. Peiffer, N. Pietsch, D. Rathjens, C. Sander, H. Schettler, P. Schleper, E. Schlieckau, A. Schmidt, M. Schröder, T. Schum, M. Seidel, J. Sibille, V. Sola, H. Stadie, G. Steinbrück, J. Thomsen, D. Troendle, E. Usai, L. Vanelderen, C. Barth, C. Baus, J. Berger, C. Böser, E. Butz, T. Chwalek, W. De Boer, A. Descroix, A. Dierlamm, M. Feindt, M. Guthoff, F. Hartmann, T. Hauth, H. Held, K. H. Hoffmann, U. Husemann, I. Katkov, J. R. Komaragiri, A. Kornmayer, P. Lobelle Pardo, D. Martschei, Th. Müller, M. Niegel, A. Nürnberg, O. Oberst, J. Ott, G. Quast, K. Rabbertz, F. Ratnikov, S. Röcker, F.-P. Schilling, G. Schott, H. J. Simonis, F. M. Stober, R. Ulrich, J. Wagner-Kuhr, S. Wayand, T. Weiler, M. Zeise, G. Anagnostou, G. Daskalakis, T. Geralis, S. Kesisoglou, A. Kyriakis, D. Loukas, A. Markou, C. Markou, E. Ntomari, L. Gouskos, A. Panagiotou, N. Saoulidou, E. Stiliaris, X. Aslanoglou, I. Evangelou, G. Flouris, C. Foudas, P. Kokkas, N. Manthos, I. Papadopoulos, E. Paradas, G. Bencze, C. Hajdu, P. Hidas, D. Horvath, F. Sikler, V. Veszpremi, G. Vesztergombi, A. J. Zsigmond, N. Beni, S. Czellar, J. Molnar, J. Palinkas, Z. Szillasi, J. Karancsi, P. Raics, Z. L. Trocsanyi, B. Ujvari, S. K. Swain, S. B. Beri, V. Bhatnagar, N. Dhingra, R. Gupta, M. Kaur, M. Z. Mehta, M. Mittal, N. Nishu, L. K. Saini, A. Sharma, J. B. Singh, Ashok Kumar, Arun Kumar, S. Ahuja, A. Bhardwaj, B. C. Choudhary, S. Malhotra, M. Naimuddin, K. Ranjan, P. Saxena, V. Sharma, R. K. Shivpuri, S. Banerjee, S. Bhattacharya, K. Chatterjee, S. Dutta, B. Gomber, Sa. Jain, Sh. Jain, R. Khurana, A. Modak, S. Mukherjee, D. Roy, S. Sarkar, M. Sharan, A. Abdulsalam, D. Dutta, S. Kailas, V. Kumar, A. K. Mohanty, L. M. Pant, P. Shukla, A. Topkar, T. Aziz, R. M. Chatterjee, S. Ganguly, S. Ghosh, M. Guchait, A. Gurtu, G. Kole, S. Kumar, M. Maity, G. Majumder, K. Mazumdar, G. B. Mohanty, B. Parida, K. Sudhakar, N. Wickramage, S. Dugad, H. Arfaei, H. Bakhshiansohi, S. M. Etesami, A. Fahim, A. Jafari, M. Khakzad, M. Mohammadi Najafabadi, S. Paktinat Mehdiabadi, B. Safarzadeh, M. Zeinali, M. Grunewald, M. Abbrescia, L. Barbone, C. Calabria, S. S. Chhibra, A. Colaleo, D. Creanza, N. De Filippis, M. De Palma, L. Fiore, G. Iaselli, G. Maggi, M. Maggi, B. Marangelli, S. My, S. Nuzzo, N. Pacifico, A. Pompili, G. Pugliese, G. Selvaggi, L. Silvestris, G. Singh, R. Venditti, P. Verwilligen, G. Zito, G. Abbiendi, A. C. Benvenuti, D. Bonacorsi, S. Braibant-Giacomelli, L. Brigliadori, R. Campanini, P. Capiluppi, A. Castro, F. R. Cavallo, G. Codispoti, M. Cuffiani, G. M. Dallavalle, F. Fabbri, A. Fanfani, D. Fasanella, P. Giacomelli, C. Grandi, L. Guiducci, S. Marcellini, G. Masetti, M. Meneghelli, A. Montanari, F. L. Navarria, F. Odorici, A. Perrotta, F. Primavera, A. M. Rossi, T. Rovelli, G. P. Siroli, N. Tosi, R. Travaglini, S. Albergo, M. Chiorboli, S. Costa, F. Giordano, R. Potenza, A. Tricomi, C. Tuve, G. Barbagli, V. Ciulli, C. Civinini, R. D’Alessandro, E. Focardi, S. Frosali, E. Gallo, S. Gonzi, V. Gori, P. Lenzi, M. Meschini, S. Paoletti, G. Sguazzoni, A. Tropiano, L. Benussi, S. Bianco, D. Piccolo, P. Fabbricatore, R. Musenich, S. Tosi, A. Benaglia, F. De Guio, M. E. Dinardo, S. Fiorendi, S. Gennai, A. Ghezzi, P. Govoni, M. T. Lucchini, S. Malvezzi, R. A. Manzoni, A. Martelli, D. Menasce, L. Moroni, M. Paganoni, D. Pedrini, S. Ragazzi, N. Redaelli, T. Tabarelli de Fatis, S. Buontempo, N. Cavallo, A. De Cosa, F. Fabozzi, A. O. M. Iorio, L. Lista, S. Meola, M. Merola, P. Paolucci, P. Azzi, N. Bacchetta, D. Bisello, A. Branca, R. Carlin, P. Checchia, T. Dorigo, U. Dosselli, M. Galanti, F. Gasparini, U. Gasparini, P. Giubilato, F. Gonella, A. Gozzelino, K. Kanishchev, S. Lacaprara, I. Lazzizzera, M. Margoni, A. T. Meneguzzo, F. Montecassiano, M. Passaseo, J. Pazzini, N. Pozzobon, P. Ronchese, F. Simonetto, E. Torassa, M. Tosi, S. Vanini, P. Zotto, A. Zucchetta, G. Zumerle, F. K. Kanishchev, M. Gabusi, S. P. Ratti, C. Riccardi, P. Vitulo, M. Biasini, G. M. Bilei, L. Fanò, P. Lariccia, G. Mantovani, M. Menichelli, A. Nappi, F. Romeo, A. Saha, A. Santocchia, A. Spiezia, K. Androsov, P. Azzurri, G. Bagliesi, J. Bernardini, T. Boccali, G. Broccolo, R. Castaldi, M. A. Ciocci, R. T. D’Agnolo, R. Dell’Orso, F. Fiori, L. Foà, A. Giassi, M. T. Grippo, A. Kraan, F. Ligabue, T. Lomtadze, L. Martini, A. Messineo, F. Palla, A. Rizzi, A. Savoy-Navarro, A. T. Serban, P. Spagnolo, P. Squillacioti, R. Tenchini, G. Tonelli, A. Venturi, P. G. Verdini, C. Vernieri, L. Barone, F. Cavallari, D. Del Re, M. Diemoz, M. Grassi, E. Longo, F. Margaroli, P. Meridiani, F. Micheli, S. Nourbakhsh, G. Organtini, R. Paramatti, S. Rahatlou, C. Rovelli, L. Soffi, N. Amapane, R. Arcidiacono, S. Argiro, M. Arneodo, R. Bellan, C. Biino, N. Cartiglia, S. Casasso, M. Costa, N. Demaria, C. Mariotti, S. Maselli, G. Mazza, E. Migliore, V. Monaco, M. Musich, M. M. Obertino, N. Pastrone, M. Pelliccioni, A. Potenza, A. Romero, M. Ruspa, R. Sacchi, A. Solano, A. Staiano, U. Tamponi, S. Belforte, V. Candelise, M. Casarsa, F. Cossutti, G. Della Ricca, B. Gobbo, C. La Licata, M. Marone, D. Montanino, A. Penzo, A. Schizzi, A. Zanetti, S. Chang, T. Y. Kim, S. K. Nam, D. H. Kim, G. N. Kim, J. E. Kim, D. J. Kong, Y. D. Oh, H. Park, D. C. Son, J. Y. Kim, Zero J. Kim, S. Song, S. Choi, D. Gyun, B. Hong, M. Jo, H. Kim, T. J. Kim, K. S. Lee, S. K. Park, Y. Roh, M. Choi, J. H. Kim, C. Park, I. C. Park, S. Park, G. Ryu, Y. Choi, Y. K. Choi, J. Goh, M. S. Kim, E. Kwon, B. Lee, J. Lee, S. Lee, H. Seo, I. Yu, I. Grigelionis, A. Juodagalvis, H. Castilla-Valdez, E. De La Cruz-Burelo, I. Heredia de La Cruz, R. Lopez-Fernandez, J. Martínez-Ortega, A. Sanchez-Hernandez, L. M. Villasenor-Cendejas, S. Carrillo Moreno, F. Vazquez Valencia, H. A. Salazar Ibarguen, E. Casimiro Linares, A. Morelos Pineda, M. A. Reyes-Santos, D. Krofcheck, A. J. Bell, P. H. Butler, R. Doesburg, S. Reucroft, H. Silverwood, M. Ahmad, M. I. Asghar, J. Butt, H. R. Hoorani, S. Khalid, W. A. Khan, T. Khurshid, S. Qazi, M. A. Shah, M. Shoaib, H. Bialkowska, B. Boimska, T. Frueboes, M. Górski, M. Kazana, K. Nawrocki, K. Romanowska-Rybinska, M. Szleper, G. Wrochna, P. Zalewski, G. Brona, K. Bunkowski, M. Cwiok, W. Dominik, K. Doroba, A. Kalinowski, M. Konecki, J. Krolikowski, M. Misiura, W. Wolszczak, N. Almeida, P. Bargassa, C. Beirão Da Cruz E Silva, P. Faccioli, P. G. Ferreira Parracho, M. Gallinaro, F. Nguyen, J. Rodrigues Antunes, J. Seixas, J. Varela, P. Vischia, S. Afanasiev, P. Bunin, M. Gavrilenko, I. Golutvin, I. Gorbunov, V. Karjavin, V. Konoplyanikov, G. Kozlov, A. Lanev, A. Malakhov, V. Matveev, P. Moisenz, V. Palichik, V. Perelygin, S. Shmatov, N. Skatchkov, V. Smirnov, A. Zarubin, S. Evstyukhin, V. Golovtsov, Y. Ivanov, V. Kim, P. Levchenko, V. Murzin, V. Oreshkin, I. Smirnov, V. Sulimov, L. Uvarov, S. Vavilov, A. Vorobyev, An. Vorobyev, Yu. Andreev, A. Dermenev, S. Gninenko, N. Golubev, M. Kirsanov, N. Krasnikov, A. Pashenkov, D. Tlisov, A. Toropin, V. Epshteyn, M. Erofeeva, V. Gavrilov, N. Lychkovskaya, V. Popov, G. Safronov, S. Semenov, A. Spiridonov, V. Stolin, E. Vlasov, A. Zhokin, V. Andreev, M. Azarkin, I. Dremin, M. Kirakosyan, A. Leonidov, G. Mesyats, S. V. Rusakov, A. Vinogradov, A. Belyaev, E. Boos, A. Ershov, A. Gribushin, V. Klyukhin, O. Kodolova, V. Korotkikh, I. Lokhtin, A. Markina, S. Obraztsov, S. Petrushanko, V. Savrin, A. Snigirev, I. Vardanyan, I. Azhgirey, I. Bayshev, S. Bitioukov, V. Kachanov, A. Kalinin, D. Konstantinov, V. Krychkine, V. Petrov, R. Ryutin, A. Sobol, L. Tourtchanovitch, S. Troshin, N. Tyurin, A. Uzunian, A. Volkov, P. Adzic, M. Djordjevic, M. Ekmedzic, D. Krpic, J. Milosevic, M. Aguilar-Benitez, J. Alcaraz Maestre, C. Battilana, E. Calvo, M. Cerrada, M. Chamizo Llatas, N. Colino, B. De La Cruz, A. Delgado Peris, D. Domínguez Vázquez, C. Fernandez Bedoya, J. P. Fernández Ramos, A. Ferrando, J. Flix, M. C. Fouz, P. Garcia-Abia, O. Gonzalez Lopez, S. Goy Lopez, J. M. Hernandez, M. I. Josa, G. Merino, E. Navarro De Martino, J. Puerta Pelayo, A. Quintario Olmeda, I. Redondo, L. Romero, J. Santaolalla, M. S. Soares, C. Willmott, C. Albajar, J. F. de Trocóniz, H. Brun, J. Cuevas, J. Fernandez Menendez, S. Folgueras, I. Gonzalez Caballero, L. Lloret Iglesias, J. Piedra Gomez, J. A. Brochero Cifuentes, I. J. Cabrillo, A. Calderon, S. H. Chuang, J. Duarte Campderros, M. Fernandez, G. Gomez, J. Gonzalez Sanchez, A. Graziano, C. Jorda, A. Lopez Virto, J. Marco, R. Marco, C. Martinez Rivero, F. Matorras, F. J. Munoz Sanchez, T. Rodrigo, A. Y. Rodríguez-Marrero, A. Ruiz-Jimeno, L. Scodellaro, I. Vila, R. Vilar Cortabitarte, D. Abbaneo, E. Auffray, G. Auzinger, M. Bachtis, P. Baillon, A. H. Ball, D. Barney, J. Bendavid, J. F. Benitez, C. Bernet, G. Bianchi, P. Bloch, A. Bocci, A. Bonato, O. Bondu, C. Botta, H. Breuker, T. Camporesi, G. Cerminara, T. Christiansen, J. A. Coarasa Perez, S. Colafranceschi, D. d’Enterria, A. Dabrowski, A. David, A. De Roeck, S. De Visscher, S. Di Guida, M. Dobson, N. Dupont-Sagorin, A. Elliott-Peisert, J. Eugster, W. Funk, G. Georgiou, M. Giffels, D. Gigi, K. Gill, D. Giordano, M. Girone, M. Giunta, F. Glege, R. Gomez-Reino Garrido, S. Gowdy, R. Guida, J. Hammer, M. Hansen, P. Harris, C. Hartl, A. Hinzmann, V. Innocente, P. Janot, E. Karavakis, K. Kousouris, K. Krajczar, P. Lecoq, Y.-J. Lee, C. Lourenço, N. Magini, M. Malberti, L. Malgeri, M. Mannelli, L. Masetti, F. Meijers, S. Mersi, E. Meschi, R. Moser, M. Mulders, P. Musella, E. Nesvold, L. Orsini, E. Palencia Cortezon, E. Perez, L. Perrozzi, A. Petrilli, A. Pfeiffer, M. Pierini, M. Pimiä, D. Piparo, M. Plagge, L. Quertenmont, A. Racz, W. Reece, G. Rolandi, M. Rovere, H. Sakulin, F. Santanastasio, C. Schäfer, C. Schwick, I. Segoni, S. Sekmen, P. Siegrist, P. Silva, M. Simon, P. Sphicas, D. Spiga, M. Stoye, A. Tsirou, G. I. Veres, J. R. Vlimant, H. K. Wöhri, S. D. Worm, W. D. Zeuner, W. Bertl, K. Deiters, W. Erdmann, K. Gabathuler, R. Horisberger, Q. Ingram, H. C. Kaestli, S. König, D. Kotlinski, U. Langenegger, D. Renker, T. Rohe, F. Bachmair, L. Bäni, L. Bianchini, P. Bortignon, M. A. Buchmann, B. Casal, N. Chanon, A. Deisher, G. Dissertori, M. Dittmar, M. Donegà, M. Dünser, P. Eller, K. Freudenreich, C. Grab, D. Hits, P. Lecomte, W. Lustermann, B. Mangano, A. C. Marini, P. Martinez Ruiz del Arbol, D. Meister, N. Mohr, F. Moortgat, C. Nägeli, P. Nef, F. Nessi-Tedaldi, F. Pandolfi, L. Pape, F. Pauss, M. Peruzzi, F. J. Ronga, M. Rossini, L. Sala, A. K. Sanchez, A. Starodumov, B. Stieger, M. Takahashi, L. Tauscher, A. Thea, K. Theofilatos, D. Treille, C. Urscheler, R. Wallny, H. A. Weber, C. Amsler, V. Chiochia, C. Favaro, M. Ivova Rikova, B. Kilminster, B. Millan Mejias, P. Otiougova, P. Robmann, H. Snoek, S. Taroni, S. Tupputi, M. Verzetti, M. Cardaci, K. H. Chen, C. Ferro, C. M. Kuo, S. W. Li, W. Lin, Y. J. Lu, R. Volpe, S. S. Yu, P. Bartalini, P. Chang, Y. H. Chang, Y. W. Chang, Y. Chao, K. F. Chen, C. Dietz, U. Grundler, W.-S. Hou, Y. Hsiung, K. Y. Kao, Y. J. Lei, R.-S. Lu, D. Majumder, E. Petrakou, X. Shi, J. G. Shiu, Y. M. Tzeng, M. Wang, B. Asavapibhop, N. Suwonjandee, A. Adiguzel, M. N. Bakirci, S. Cerci, C. Dozen, I. Dumanoglu, E. Eskut, S. Girgis, G. Gokbulut, E. Gurpinar, I. Hos, E. E. Kangal, A. Kayis Topaksu, G. Onengut, K. Ozdemir, S. Ozturk, A. Polatoz, K. Sogut, D. Sunar Cerci, B. Tali, H. Topakli, M. Vergili, I. V. Akin, T. Aliev, B. Bilin, S. Bilmis, M. Deniz, H. Gamsizkan, A. M. Guler, G. Karapinar, K. Ocalan, A. Ozpineci, M. Serin, R. Sever, U. E. Surat, M. Yalvac, M. Zeyrek, E. Gülmez, B. Isildak, M. Kaya, O. Kaya, S. Ozkorucuklu, N. Sonmez, H. Bahtiyar, E. Barlas, K. Cankocak, Y. O. Günaydin, F. I. Vardarlı, M. Yücel, L. Levchuk, P. Sorokin, J. J. Brooke, E. Clement, D. Cussans, H. Flacher, R. Frazier, J. Goldstein, M. Grimes, G. P. Heath, H. F. Heath, L. Kreczko, S. Metson, D. M. Newbold, K. Nirunpong, A. Poll, S. Senkin, V. J. Smith, T. Williams, A. Belyaev, C. Brew, R. M. Brown, D. J. A. Cockerill, J. A. Coughlan, K. Harder, S. Harper, E. Olaiya, D. Petyt, B. C. Radburn-Smith, C. H. Shepherd-Themistocleous, I. R. Tomalin, W. J. Womersley, R. Bainbridge, O. Buchmuller, D. Burton, D. Colling, N. Cripps, M. Cutajar, P. Dauncey, G. Davies, M. Della Negra, W. Ferguson, J. Fulcher, D. Futyan, A. Gilbert, A. Guneratne Bryer, G. Hall, Z. Hatherell, J. Hays, G. Iles, M. Jarvis, G. Karapostoli, M. Kenzie, R. Lane, R. Lucas, L. Lyons, A.-M. Magnan, J. Marrouche, B. Mathias, R. Nandi, J. Nash, A. Nikitenko, J. Pela, M. Pesaresi, K. Petridis, M. Pioppi, D. M. Raymond, S. Rogerson, A. Rose, C. Seez, P. Sharp, A. Sparrow, A. Tapper, M. Vazquez Acosta, T. Virdee, S. Wakefield, N. Wardle, T. Whyntie, M. Chadwick, J. E. Cole, P. R. Hobson, A. Khan, P. Kyberd, D. Leggat, D. Leslie, W. Martin, I. D. Reid, P. Symonds, L. Teodorescu, M. Turner, J. Dittmann, K. Hatakeyama, A. Kasmi, H. Liu, T. Scarborough, O. Charaf, S. I. Cooper, C. Henderson, P. Rumerio, A. Avetisyan, T. Bose, C. Fantasia, A. Heister, P. Lawson, D. Lazic, J. Rohlf, D. Sperka, J. St. John, L. Sulak, J. Alimena, G. Christopher, D. Cutts, Z. Demiragli, A. Ferapontov, A. Garabedian, U. Heintz, S. Jabeen, G. Kukartsev, E. Laird, G. Landsberg, M. Luk, M. Narain, M. Segala, T. Sinthuprasith, T. Speer, R. Breedon, G. Breto, M. Calderon De La Barca Sanchez, S. Chauhan, M. Chertok, J. Conway, R. Conway, P. T. Cox, R. Erbacher, M. Gardner, R. Houtz, W. Ko, A. Kopecky, R. Lander, T. Miceli, D. Pellett, F. Ricci-Tam, B. Rutherford, M. Searle, J. Smith, M. Squires, M. Tripathi, S. Wilbur, R. Yohay, V. Andreev, D. Cline, R. Cousins, S. Erhan, P. Everaerts, C. Farrell, M. Felcini, J. Hauser, M. Ignatenko, C. Jarvis, G. Rakness, P. Schlein, E. Takasugi, P. Traczyk, V. Valuev, J. Babb, R. Clare, J. Ellison, J. W. Gary, G. Hanson, P. Jandir, H. Liu, O. R. Long, A. Luthra, H. Nguyen, S. Paramesvaran, J. Sturdy, S. Sumowidagdo, R. Wilken, S. Wimpenny, W. Andrews, J. G. Branson, G. B. Cerati, S. Cittolin, D. Evans, A. Holzner, R. Kelley, M. Lebourgeois, J. Letts, I. Macneill, S. Padhi, C. Palmer, G. Petrucciani, M. Pieri, M. Sani, S. Simon, E. Sudano, M. Tadel, Y. Tu, A. Vartak, S. Wasserbaech, F. Würthwein, A. Yagil, J. Yoo, D. Barge, C. Campagnari, M. D’Alfonso, T. Danielson, K. Flowers, P. Geffert, C. George, F. Golf, J. Incandela, C. Justus, P. Kalavase, D. Kovalskyi, V. Krutelyov, S. Lowette, R. Magaña Villalba, N. Mccoll, V. Pavlunin, J. Ribnik, J. Richman, R. Rossin, D. Stuart, W. To, C. West, A. Apresyan, A. Bornheim, J. Bunn, Y. Chen, E. Di Marco, J. Duarte, D. Kcira, Y. Ma, A. Mott, H. B. Newman, C. Rogan, M. Spiropulu, V. Timciuc, J. Veverka, R. Wilkinson, S. Xie, Y. Yang, R. Y. Zhu, V. Azzolini, A. Calamba, R. Carroll, T. Ferguson, Y. Iiyama, D. W. Jang, Y. F. Liu, M. Paulini, J. Russ, H. Vogel, I. Vorobiev, J. P. Cumalat, B. R. Drell, W. T. Ford, A. Gaz, E. Luiggi Lopez, U. Nauenberg, J. G. Smith, K. Stenson, K. A. Ulmer, S. R. Wagner, J. Alexander, A. Chatterjee, N. Eggert, L. K. Gibbons, W. Hopkins, A. Khukhunaishvili, B. Kreis, N. Mirman, G. Nicolas Kaufman, J. R. Patterson, A. Ryd, E. Salvati, W. Sun, W. D. Teo, J. Thom, J. Thompson, J. Tucker, Y. Weng, L. Winstrom, P. Wittich, D. Winn, S. Abdullin, M. Albrow, J. Anderson, G. Apollinari, L. A. T. Bauerdick, A. Beretvas, J. Berryhill, P. C. Bhat, K. Burkett, J. N. Butler, V. Chetluru, H. W. K. Cheung, F. Chlebana, S. Cihangir, V. D. Elvira, I. Fisk, J. Freeman, Y. Gao, E. Gottschalk, L. Gray, D. Green, O. Gutsche, D. Hare, R. M. Harris, J. Hirschauer, B. Hooberman, S. Jindariani, M. Johnson, U. Joshi, K. Kaadze, B. Klima, S. Kunori, S. Kwan, J. Linacre, D. Lincoln, R. Lipton, J. Lykken, K. Maeshima, J. M. Marraffino, V. I. Martinez Outschoorn, S. Maruyama, D. Mason, P. McBride, K. Mishra, S. Mrenna, Y. Musienko, C. Newman-Holmes, V. O’Dell, O. Prokofyev, N. Ratnikova, E. Sexton-Kennedy, S. Sharma, W. J. Spalding, L. Spiegel, L. Taylor, S. Tkaczyk, N. V. Tran, L. Uplegger, E. W. Vaandering, R. Vidal, J. Whitmore, W. Wu, F. Yang, J. C. Yun, D. Acosta, P. Avery, D. Bourilkov, M. Chen, T. Cheng, S. Das, M. De Gruttola, G. P. Di Giovanni, D. Dobur, A. Drozdetskiy, R. D. Field, M. Fisher, Y. Fu, I. K. Furic, J. Hugon, B. Kim, J. Konigsberg, A. Korytov, A. Kropivnitskaya, T. Kypreos, J. F. Low, K. Matchev, P. Milenovic, G. Mitselmakher, L. Muniz, R. Remington, A. Rinkevicius, N. Skhirtladze, M. Snowball, J. Yelton, M. Zakaria, V. Gaultney, S. Hewamanage, S. Linn, P. Markowitz, G. Martinez, J. L. Rodriguez, T. Adams, A. Askew, J. Bochenek, J. Chen, B. Diamond, S. V. Gleyzer, J. Haas, S. Hagopian, V. Hagopian, K. F. Johnson, H. Prosper, V. Veeraraghavan, M. Weinberg, M. M. Baarmand, B. Dorney, M. Hohlmann, H. Kalakhety, F. Yumiceva, M. R. Adams, L. Apanasevich, V. E. Bazterra, R. R. Betts, I. Bucinskaite, J. Callner, R. Cavanaugh, O. Evdokimov, L. Gauthier, C. E. Gerber, D. J. Hofman, S. Khalatyan, P. Kurt, F. Lacroix, D. H. Moon, C. O’Brien, C. Silkworth, D. Strom, P. Turner, N. Varelas, U. Akgun, E. A. Albayrak, B. Bilki, W. Clarida, K. Dilsiz, F. Duru, S. Griffiths, J.-P. Merlo, H. Mermerkaya, A. Mestvirishvili, A. Moeller, J. Nachtman, C. R. Newsom, H. Ogul, Y. Onel, F. Ozok, S. Sen, P. Tan, E. Tiras, J. Wetzel, T. Yetkin, K. Yi, B. A. Barnett, B. Blumenfeld, S. Bolognesi, G. Giurgiu, A. V. Gritsan, G. Hu, P. Maksimovic, C. Martin, M. Swartz, A. Whitbeck, P. Baringer, A. Bean, G. Benelli, R. P. Kenny, M. Murray, D. Noonan, S. Sanders, R. Stringer, J. S. Wood, A. F. Barfuss, I. Chakaberia, A. Ivanov, S. Khalil, M. Makouski, Y. Maravin, S. Shrestha, I. Svintradze, J. Gronberg, D. Lange, F. Rebassoo, D. Wright, A. Baden, B. Calvert, S. C. Eno, J. A. Gomez, N. J. Hadley, R. G. Kellogg, T. Kolberg, Y. Lu, M. Marionneau, A. C. Mignerey, K. Pedro, A. Peterman, A. Skuja, J. Temple, M. B. Tonjes, S. C. Tonwar, A. Apyan, G. Bauer, W. Busza, I. A. Cali, M. Chan, L. Di Matteo, V. Dutta, G. Gomez Ceballos, M. Goncharov, D. Gulhan, Y. Kim, M. Klute, Y. S. Lai, A. Levin, P. D. Luckey, T. Ma, S. Nahn, C. Paus, D. Ralph, C. Roland, G. Roland, G. S. F. Stephans, F. Stöckli, K. Sumorok, D. Velicanu, R. Wolf, B. Wyslouch, M. Yang, Y. Yilmaz, A. S. Yoon, M. Zanetti, V. Zhukova, B. Dahmes, A. De Benedetti, G. Franzoni, A. Gude, J. Haupt, S. C. Kao, K. Klapoetke, Y. Kubota, J. Mans, N. Pastika, R. Rusack, M. Sasseville, A. Singovsky, N. Tambe, J. Turkewitz, J. G. Acosta, L. M. Cremaldi, R. Kroeger, S. Oliveros, L. Perera, R. Rahmat, D. A. Sanders, D. Summers, E. Avdeeva, K. Bloom, S. Bose, D. R. Claes, A. Dominguez, M. Eads, R. Gonzalez Suarez, J. Keller, I. Kravchenko, J. Lazo-Flores, S. Malik, F. Meier, G. R. Snow, J. Dolen, A. Godshalk, I. Iashvili, S. Jain, A. Kharchilava, A. Kumar, S. Rappoccio, Z. Wan, G. Alverson, E. Barberis, D. Baumgartel, M. Chasco, J. Haley, A. Massironi, D. Nash, T. Orimoto, D. Trocino, D. Wood, J. Zhang, A. Anastassov, K. A. Hahn, A. Kubik, L. Lusito, N. Mucia, N. Odell, B. Pollack, A. Pozdnyakov, M. Schmitt, S. Stoynev, K. Sung, M. Velasco, S. Won, D. Berry, A. Brinkerhoff, K. M. Chan, M. Hildreth, C. Jessop, D. J. Karmgard, J. Kolb, K. Lannon, W. Luo, S. Lynch, N. Marinelli, D. M. Morse, T. Pearson, M. Planer, R. Ruchti, J. Slaunwhite, N. Valls, M. Wayne, M. Wolf, L. Antonelli, B. Bylsma, L. S. Durkin, C. Hill, R. Hughes, K. Kotov, T. Y. Ling, D. Puigh, M. Rodenburg, G. Smith, C. Vuosalo, B. L. Winer, H. Wolfe, E. Berry, P. Elmer, V. Halyo, P. Hebda, J. Hegeman, A. Hunt, P. Jindal, S. A. Koay, P. Lujan, D. Marlow, T. Medvedeva, M. Mooney, J. Olsen, P. Piroué, X. Quan, A. Raval, H. Saka, D. Stickland, C. Tully, J. S. Werner, S. C. Zenz, A. Zuranski, E. Brownson, A. Lopez, H. Mendez, J. E. Ramirez Vargas, E. Alagoz, D. Benedetti, G. Bolla, D. Bortoletto, M. De Mattia, A. Everett, Z. Hu, M. Jones, K. Jung, O. Koybasi, M. Kress, N. Leonardo, D. Lopes Pegna, V. Maroussov, P. Merkel, D. H. Miller, N. Neumeister, I. Shipsey, D. Silvers, A. Svyatkovskiy, M. Vidal Marono, F. Wang, W. Xie, L. Xu, H. D. Yoo, J. Zablocki, Y. Zheng, S. Guragain, N. Parashar, A. Adair, B. Akgun, K. M. Ecklund, F. J. M. Geurts, B. P. Padley, R. Redjimi, J. Roberts, J. Zabel, B. Betchart, A. Bodek, R. Covarelli, P. de Barbaro, R. Demina, Y. Eshaq, T. Ferbel, A. Garcia-Bellido, P. Goldenzweig, J. Han, A. Harel, D. C. Miner, G. Petrillo, D. Vishnevskiy, M. Zielinski, A. Bhatti, R. Ciesielski, L. Demortier, K. Goulianos, G. Lungu, S. Malik, C. Mesropian, S. Arora, A. Barker, J. P. Chou, C. Contreras-Campana, E. Contreras-Campana, D. Duggan, D. Ferencek, Y. Gershtein, R. Gray, E. Halkiadakis, D. Hidas, A. Lath, S. Panwalkar, M. Park, R. Patel, V. Rekovic, J. Robles, S. Salur, S. Schnetzer, C. Seitz, S. Somalwar, R. Stone, S. Thomas, P. Thomassen, M. Walker, G. Cerizza, M. Hollingsworth, K. Rose, S. Spanier, Z. C. Yang, A. York, O. Bouhali, R. Eusebi, W. Flanagan, J. Gilmore, T. Kamon, V. Khotilovich, R. Montalvo, I. Osipenkov, Y. Pakhotin, A. Perloff, J. Roe, A. Safonov, T. Sakuma, I. Suarez, A. Tatarinov, D. Toback, N. Akchurin, C. Cowden, J. Damgov, C. Dragoiu, P. R. Dudero, C. Jeong, K. Kovitanggoon, S. W. Lee, T. Libeiro, I. Volobouev, E. Appelt, A. G. Delannoy, S. Greene, A. Gurrola, W. Johns, C. Maguire, A. Melo, M. Sharma, P. Sheldon, B. Snook, S. Tuo, J. Velkovska, M. W. Arenton, S. Boutle, B. Cox, B. Francis, J. Goodell, R. Hirosky, A. Ledovskoy, C. Lin, C. Neu, J. Wood, S. Gollapinni, R. Harr, P. E. Karchin, C. Kottachchi Kankanamge Don, P. Lamichhane, A. Sakharov, D. A. Belknap, L. Borrello, D. Carlsmith, M. Cepeda, S. Dasu, E. Friis, M. Grothe, R. Hall-Wilton, M. Herndon, A. Hervé, P. Klabbers, J. Klukas, A. Lanaro, R. Loveless, A. Mohapatra, M. U. Mozer, I. Ojalvo, G. A. Pierro, G. Polese, I. Ross, A. Savin, W. H. Smith, J. Swanson

**Affiliations:** 1 Yerevan Physics Institute, Yerevan, Armenia; 2 Institut für Hochenergiephysik der OeAW, Wien, Austria; 3 National Centre for Particle and High Energy Physics, Minsk, Belarus; 4 Universiteit Antwerpen, Antwerpen, Belgium; 5 Vrije Universiteit Brussel, Brussel, Belgium; 6 Université Libre de Bruxelles, Bruxelles, Belgium; 7 Ghent University, Ghent, Belgium; 8 Université Catholique de Louvain, Louvain-la-Neuve, Belgium; 9 Université de Mons, Mons, Belgium; 10 Centro Brasileiro de Pesquisas Fisicas, Rio de Janeiro, Brazil; 11 Universidade do Estado do Rio de Janeiro, Rio de Janeiro, Brazil; 12 Universidade Estadual Paulista, São Paulo, Brazil; 13 Universidade Federal do ABC, São Paulo, Brazil; 14 Institute for Nuclear Research and Nuclear Energy, Sofia, Bulgaria; 15 University of Sofia, Sofia, Bulgaria; 16 Institute of High Energy Physics, Beijing, China; 17 State Key Laboratory of Nuclear Physics and Technology, Peking University, Beijing, China; 18 Universidad de Los Andes, Bogota, Colombia; 19 Technical University of Split, Split, Croatia; 20 University of Split, Split, Croatia; 21 Institute Rudjer Boskovics, Zagreb, Croatia; 22 University of Cyprus, Nicosia, Cyprus; 23 Charles University, Prague, Czech Republic; 24 Academy of Scientific Research and Technology of the Arab Republic of Egypt, Egyptian Network of High Energy Physics, Cairo, Egypt; 25 National Institute of Chemical Physics and Biophysics, Tallinn, Estonia; 26 Department of Physics, University of Helsinki, Helsinki, Finland; 27 Helsinki Institute of Physics, Helsinki, Finland; 28 Lappeenranta University of Technology, Lappeenranta, Finland; 29 DSM/IRFU, CEA/Saclay, Gif-sur-Yvette, France; 30 Laboratoire Leprince-Ringuet, Ecole Polytechnique, IN2P3-CNRS Palaiseau, France; 31 Institut Pluridisciplinaire Hubert Curien Université de Strasbourg, Université de Haute Alsace Mulhouse, CNRS/IN2P3 Strasbourg, France; 32 Centre de Calcul de l’Institut National de Physique Nucleaire et de Physique des Particules, CNRS/IN2P3 Villeurbanne, France; 33 Université de Lyon, Université Claude Bernard Lyon 1, CNRS-IN2P3, Institut de Physique Nucléaire de Lyon, Villeurbanne, France; 34 Institute of High Energy Physics and Informatization, Tbilisi State University, Tbilisi, Georgia; 35 RWTH Aachen University, I. Physikalisches Institut, Aachen, Germany; 36 RWTH Aachen University, III. Physikalisches Institut A, Aachen, Germany; 37 RWTH Aachen University, III. Physikalisches Institut B, Aachen, Germany; 38 Deutsches Elektronen-Synchrotron, Hamburg, Germany; 39 University of Hamburg, Hamburg, Germany; 40 Institut für Experimentelle Kernphysik, Karlsruhe, Germany; 41 Institute of Nuclear and Particle Physics (INPP), NCSR Demokritos, Aghia Paraskevi, Greece; 42 University of Athens, Athens, Greece; 43 University of Ioánnina, Ioánnina, Greece; 44 KFKI Research Institute for Particle and Nuclear Physics, Budapest, Hungary; 45 Institute of Nuclear Research ATOMKI, Debrecen, Hungary; 46 University of Debrecen, Debrecen, Hungary; 47 National Institute of Science Education and Research, Bhubaneswar, India; 48 Panjab University, Chandigarh, India; 49 University of Delhi, Delhi, India; 50 Saha Institute of Nuclear Physics, Kolkata, India; 51 Bhabha Atomic Research Centre, Mumbai, India; 52 Tata Institute of Fundamental Research - EHEP, Mumbai, India; 53 Tata Institute of Fundamental Research - HECR, Mumbai, India; 54 Institute for Research in Fundamental Sciences (IPM), Tehran, Iran; 55 University College Dublin, Dublin, Ireland; 56 INFN Sezione di Bari, Bari, Italy; 57 Università di Bari, Bari, Italy; 58 Politecnico di Bari, Bari, Italy; 59 INFN Sezione di Bologna, Bologna, Italy; 60 Università di Bologna, Bologna, Italy; 61 INFN Sezione di Catania, Catania, Italy; 62 Università di Catania, Catania, Italy; 63 INFN Sezione di Firenze, Firenze, Italy; 64 Università di Firenze, Firenze, Italy; 65 INFN Laboratori Nazionali di Frascati, Frascati, Italy; 66 INFN Sezione di Genova, Genova, Italy; 67 Università di Genova, Genova, Italy; 68 INFN Sezione di Milano-Bicocca, Milano, Italy; 69 Università di Milano-Bicocca, Milano, Italy; 70 INFN Sezione di Napoli, Napoli, Italy; 71 Università di Napoli ’Federico II’, Napoli, Italy; 72 Università della Basilicata (Potenza), Napoli, Italy; 73 Università G. Marconi (Roma), Napoli, Italy; 74 INFN Sezione di Padova, Padova, Italy; 75 Università di Padova, Padova, Italy; 76 Università di Trento (Trento), Padova, Italy; 77 INFN Sezione di Pavia, Pavia, Italy; 78 Università di Pavia, Pavia, Italy; 79 INFN Sezione di Perugia, Perugia, Italy; 80 Università di Perugia, Perugia, Italy; 81 INFN Sezione di Pisa, Pisa, Italy; 82 Università di Pisa, Pisa, Italy; 83 Scuola Normale Superiore di Pisa, Pisa, Italy; 84 INFN Sezione di Roma, Roma, Italy; 85 Università di Roma, Roma, Italy; 86 INFN Sezione di Torino, Torino, Italy; 87 Università di Torino, Torino, Italy; 88 Università del Piemonte Orientale (Novara), Torino, Italy; 89 INFN Sezione di Trieste, Trieste, Italy; 90 Università di Trieste, Trieste, Italy; 91 Kangwon National University, Chunchon, Korea; 92 Kyungpook National University, Daegu, Korea; 93 Institute for Universe and Elementary Particles, Chonnam National University, Kwangju, Korea; 94 Korea University, Seoul, Korea; 95 University of Seoul, Seoul, Korea; 96 Sungkyunkwan University, Suwon, Korea; 97 Vilnius University, Vilnius, Lithuania; 98 Centro de Investigacion y de Estudios Avanzados del IPN, Mexico City, Mexico; 99 Universidad Iberoamericana, Mexico City, Mexico; 100 Benemerita Universidad Autonoma de Puebla, Puebla, Mexico; 101 Universidad Autónoma de San Luis Potosí, San Luis Potosí, Mexico; 102 University of Auckland, Auckland, New Zealand; 103 University of Canterbury, Christchurch, New Zealand; 104 National Centre for Physics, Quaid-I-Azam University, Islamabad, Pakistan; 105 National Centre for Nuclear Research, Swierk, Poland; 106 Institute of Experimental Physics, Faculty of Physics, University of Warsaw, Warsaw, Poland; 107 Laboratório de Instrumentação e Física Experimental de Partículas, Lisboa, Portugal; 108 Joint Institute for Nuclear Research, Dubna, Russia; 109 Petersburg Nuclear Physics Institute, Gatchina (St. Petersburg), Russia; 110 Institute for Nuclear Research, Moscow, Russia; 111 Institute for Theoretical and Experimental Physics, Moscow, Russia; 112 P.N. Lebedev Physical Institute, Moscow, Russia; 113 Skobeltsyn Institute of Nuclear Physics, Lomonosov Moscow State University, Moscow, Russia; 114 State Research Center of Russian Federation, Institute for High Energy Physics, Protvino, Russia; 115 University of Belgrade, Faculty of Physics and Vinca Institute of Nuclear Sciences, Belgrade, Serbia; 116 Centro de Investigaciones Energéticas Medioambientales y Tecnológicas (CIEMAT), Madrid, Spain; 117 Universidad Autónoma de Madrid, Madrid, Spain; 118 Universidad de Oviedo, Oviedo, Spain; 119 Instituto de Física de Cantabria (IFCA), CSIC-Universidad de Cantabria, Santander, Spain; 120 CERN, European Organization for Nuclear Research, Geneva, Switzerland; 121 Paul Scherrer Institut, Villigen, Switzerland; 122 Institute for Particle Physics, ETH Zurich, Zurich, Switzerland; 123 Universität Zürich, Zurich, Switzerland; 124 National Central University, Chung-Li, Taiwan; 125 National Taiwan University (NTU), Taipei, Taiwan; 126 Chulalongkorn University, Bangkok, Thailand; 127 Cukurova University, Adana, Turkey; 128 Physics Department, Middle East Technical University, Ankara, Turkey; 129 Bogazici University, Istanbul, Turkey; 130 Istanbul Technical University, Istanbul, Turkey; 131 National Scientific Center, Kharkov Institute of Physics and Technology, Kharkov, Ukraine; 132 University of Bristol, Bristol, UK; 133 Rutherford Appleton Laboratory, Didcot, UK; 134 Imperial College, London, UK; 135 Brunel University, Uxbridge, UK; 136 Baylor University, Waco, USA; 137 The University of Alabama, Tuscaloosa, USA; 138 Boston University, Boston, USA; 139 Brown University, Providence, USA; 140 University of California, Davis, USA; 141 University of California, Los Angeles, USA; 142 University of California, Riverside, USA; 143 University of California, San Diego, La Jolla, USA; 144 University of California, Santa Barbara, Santa Barbara, USA; 145 California Institute of Technology, Pasadena, USA; 146 Carnegie Mellon University, Pittsburgh, USA; 147 University of Colorado at Boulder, Boulder, USA; 148 Cornell University, Ithaca, USA; 149 Fairfield University, Fairfield, USA; 150 Fermi National Accelerator Laboratory, Batavia, USA; 151 University of Florida, Gainesville, USA; 152 Florida International University, Miami, USA; 153 Florida State University, Tallahassee, USA; 154 Florida Institute of Technology, Melbourne, USA; 155 University of Illinois at Chicago (UIC), Chicago, USA; 156 The University of Iowa, Iowa City, USA; 157 Johns Hopkins University, Baltimore, USA; 158 The University of Kansas, Lawrence, USA; 159 Kansas State University, Manhattan, USA; 160 Lawrence Livermore National Laboratory, Livermore, USA; 161 University of Maryland, College Park, USA; 162 Massachusetts Institute of Technology, Cambridge, USA; 163 University of Minnesota, Minneapolis, USA; 164 University of Mississippi, Oxford, USA; 165 University of Nebraska-Lincoln, Lincoln, USA; 166 State University of New York at Buffalo, Buffalo, USA; 167 Northeastern University, Boston, USA; 168 Northwestern University, Evanston, USA; 169 University of Notre Dame, Notre Dame, USA; 170 The Ohio State University, Columbus, USA; 171 Princeton University, Princeton, USA; 172 University of Puerto Rico, Mayaguez, USA; 173 Purdue University, West Lafayette, USA; 174 Purdue University Calumet, Hammond, USA; 175 Rice University, Houston, USA; 176 University of Rochester, Rochester, USA; 177 The Rockefeller University, New York, USA; 178 Rutgers, The State University of New Jersey, Piscataway, USA; 179 University of Tennessee, Knoxville, USA; 180 Texas A&M University, College Station, USA; 181 Texas Tech University, Lubbock, USA; 182 Vanderbilt University, Nashville, USA; 183 University of Virginia, Charlottesville, USA; 184 Wayne State University, Detroit, USA; 185 University of Wisconsin, Madison, USA; 186 CERN, Geneva, Switzerland

## Abstract

Spectra of identified charged hadrons are measured in pPb collisions with the CMS detector at the LHC at $\sqrt{s_{NN}} =5.02\,\text {TeV}$. Charged pions, kaons, and protons in the transverse-momentum range $p_{\mathrm {T}} \approx 0.1$–1.7${\,\text {GeV/}c}$ and laboratory rapidity $|y | < 1$ are identified via their energy loss in the silicon tracker. The average $p_{\mathrm {T}}$ increases with particle mass and the charged multiplicity of the event. The increase of the average $p_{\mathrm {T}}$ with charged multiplicity is greater for heavier hadrons. Comparisons to Monte Carlo event generators reveal that Epos Lhc, which incorporates additional hydrodynamic evolution of the created system, is able to reproduce most of the data features, unlike Hijing and Ampt. The $p_{\mathrm {T}}$ spectra and integrated yields are also compared to those measured in pp and PbPb collisions at various energies. The average transverse momentum and particle ratio measurements indicate that particle production at LHC energies is strongly correlated with event particle multiplicity.

## Introduction

The study of hadron production has a long history in high-energy particle and nuclear physics, as well as in cosmic-ray physics. The absolute yields and the transverse momentum ($p_{\mathrm {T}}$) spectra of identified hadrons in high-energy hadron–hadron collisions are among the most basic physical observables. They can be used to test the predictions for non-perturbative quantum chromodynamics (QCD) processes like hadronization and soft-parton interactions, and the validity of their implementation in Monte Carlo (MC) event generators. Spectra of identified particles in proton–nucleus collisions also constitute an important reference for studies of high-energy heavy-ion collisions, where final-state effects are known to modify the spectral shape and yields of different hadron species [1–7].

The present analysis focuses on the measurement of the $p_{\mathrm {T}}$ spectra of charged hadrons, identified mostly via their energy deposits in silicon detectors, in pPb collisions at $\sqrt{s_{NN}} =$ 5.02$\,\text {TeV}$. The analysis procedures are similar to those previously used in the measurement of pion, kaon, and proton production in pp collisions at several center-of-mass energies [8]. Results on ${\pi }$, $\mathrm {K}$, and $\mathrm {p}$ production in pPb collisions have been also reported by the ALICE Collaboration [9].

A detailed description of the CMS (Compact Muon Solenoid) detector can be found in Ref. [10]. The CMS experiment uses a right-handed coordinate system, with the origin at the nominal interaction point (IP) and the $z$ axis along the counterclockwise-beam direction. The pseudorapidity $\eta$ and rapidity $y$ of a particle (in the laboratory frame) with energy $E$, momentum $p$, and momentum along the $z$ axis $p_z$ are defined as $\eta = -\ln [\tan (\theta /2)]$, where $\theta$ is the polar angle with respect to the $z$ axis and $y = \frac{1}{2}\ln [(E+p_z)/(E-p_z)]$, respectively. The central feature of the CMS apparatus is a superconducting solenoid of 6$\,\text {m}$ internal diameter. Within the 3.8 T field volume are the silicon pixel and strip tracker, the crystal electromagnetic calorimeter, and the brass/scintillator hadron calorimeter. The tracker measures charged particles within the pseudorapidity range $|\eta | < 2.4$. It has 1440 silicon pixel and 15 148 silicon strip detector modules, ordered in 13 tracking layers in the $y$ region studied here. In addition to the barrel and endcap detectors, CMS has extensive forward calorimetry. Steel/quartz-fiber forward calorimeters (HF) cover $3 < |\eta | < 5$. Beam Pick-up Timing for the eXperiments (BPTX) devices were used to trigger the detector readout. They are located around the beam pipe at a distance of 175$\,\text {m}$ from the IP on either side, and are designed to provide precise information on the Large Hadron Collider (LHC) bunch structure and timing of the incoming beams.

The reconstruction of charged particles in CMS is bounded by the acceptance of the tracker ($|\eta | <$ 2.4) and by the decreasing tracking efficiency at low momentum (greater than about 60 % for $p > 0.05$, 0.10, 0.20, and 0.40${\,\text {GeV/}c}$ for $\mathrm {e}$, ${\pi }$, $\mathrm {K}$, and $\mathrm {p}$, respectively). Particle identification capabilities using specific ionization are restricted to $p < 0.15{\,\text {GeV/}c}$ for electrons, $p < 1.20{\,\text {GeV/}c}$ for pions, $p < 1.05{\,\text {GeV/}c}$ for kaons, and $p < 1.70{\,\text {GeV/}c}$ for protons. Pions are identified up to a higher momentum than kaons because of their high relative abundance. In view of the $(y,p_{\mathrm {T}})$ regions where pions, kaons, and protons can all be identified ($p = p_{\mathrm {T}} \cosh y$), the band $-1 < y < 1$ (in the laboratory frame) was chosen for this measurement, since it is a good compromise between the $p_{\mathrm {T}}$ range and $y$ coverage.

In this paper, comparisons are made to predictions from three MC event generators. The Hijing [11] event generator is based on a two-component model for hadron production in high-energy nucleon and nuclear collisions. Hard parton scatterings are assumed to be described by perturbative QCD and soft interactions are approximated by string excitations with an effective cross section. In version 2.1 [12], in addition to modification of initial parton distributions, multiple scatterings inside a nucleus lead to transverse momentum broadening of both initial and final-state partons. This is responsible for the enhancement of intermediate-$p_{\mathrm {T}}$ (2–6${\,\text {GeV/}c}$) hadron spectra in proton–nucleus collisions, with respect to the properly scaled spectra of proton–proton collisions (Cronin effect). The Ampt [13] event generator is a multi-phase transport model. It starts from the same initial conditions as Hijing, contains a partonic transport phase, the description of the bulk hadronization, and finally a hadronic rescattering phase. These processes lead to hydrodynamic-like effects in simulated nucleus–nucleus collisions, but not necessarily in proton–nucleus collisions. The latest available version (1.26/2.26) is used. The Epos [14] event generator uses a quantum mechanical multiple scattering approach based on partons and strings, where cross sections and particle production are calculated consistently, taking into account energy conservation in both cases. Nuclear effects related to transverse momentum broadening, parton saturation, and screening have been introduced. The model can be used both for extensive air shower simulations and accelerator physics. Epos Lhc [15] is an improvement of version 1.99 (v3400) and contains a three-dimensional viscous event-by-event hydrodynamic treatment. This is a major difference with respect to the Hijing and Ampt models for proton–nucleus collisions.

## Data analysis

The data were taken in September 2012 during a 4-h-long pPb run with very low probability of multiple interactions (0.15 % “pileup”). A total of 2.0 million collisions were collected, corresponding to an integrated luminosity of approximately $1\,\upmu \mathrm {b}^{-1}$. The dominant uncertainty for the reported measurements is systematic in nature. The beam energies were 4$\,\text {TeV}$ for protons and 1.58$\,\text {TeV}$ per nucleon for lead nuclei, resulting in a center-of-mass energy per nucleon pair of $\sqrt{s_{NN}} =$ 5.02$\,\text {TeV}$. Due to the asymmetric beam energies the nucleon-nucleon center-of-mass in the pPb collisions was not at rest with respect to the laboratory frame but was moving with a velocity $\beta = -0.434$ or rapidity $-0.465$. Since the higher-energy proton beam traveled in the clockwise direction, i.e. at $\theta = \pi$, the rapidity of a particle emitted at $y_\mathrm{cm}$ in the nucleon-nucleon center-of-mass frame is detected in the laboratory frame with a shift, $y - y_\mathrm{cm} = -0.465$, i.e. a particle with $y=0$ moves with rapidity 0.465 in the Pb-beam direction in the center-of-mass system. The particle yields reported in this paper have been measured for laboratory rapidity $|y | < 1$ to match the experimentally accessible region.

The event selection consisted of the following requirements:

- at the trigger level, the coincidence of signals from both BPTX devices, indicating the presence of both proton and lead bunches crossing the interaction point; in addition, at least one track with $p_{\mathrm {T}} > 0.4{\,\text {GeV/}c}$ in the pixel tracker;
- offline, the presence of at least one tower with energy above 3$\,\text {GeV}$ in each of the HF calorimeters; at least one reconstructed interaction vertex; beam-halo and beam-induced background events, which usually produce an anomalously large number of pixel hits [16], are suppressed.

The efficiencies for event selection, tracking, and vertexing were evaluated using simulated event samples produced with the Hijing 2.1 MC event generator, where the CMS detector response simulation was based on Geant4 [17]. Simulated events were reconstructed in the same way as collision data events. The final results were corrected to a particle level selection applied to the direct MC output, which is very similar to the data selection described above: at least one particle (proper lifetime $\tau > 10^{-18}\,\text {s}$) with $E > 3\,\text {GeV}$ in the range $-5 < \eta < -3$ and at least one in the range $3 < \eta <5$; this selection is referred to in the following as the “double-sided” (DS) selection. These requirements are expected to suppress single-diffractive collisions in both the data and MC samples. From the MC event generators studied in this paper, the DS selection efficiency for inelastic, hadronic collisions is found to be 94–97 %.

**Fig. 1:**
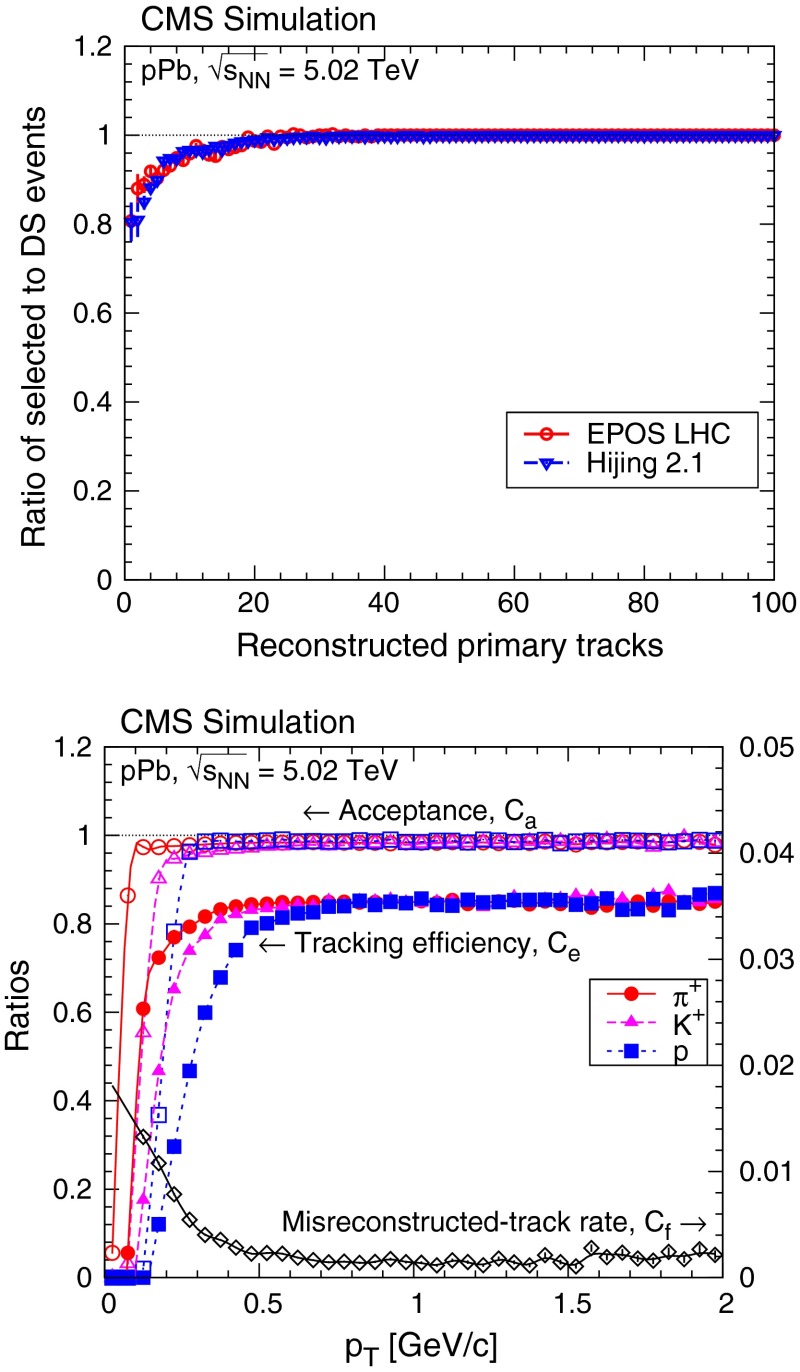
*Top* the ratio of selected events to double-sided (DS) events (ratio of the corresponding efficiencies in the inelastic sample), according to Epos Lhc and Hijing MC simulations, as a function of the reconstructed primary charged-particle multiplicity. *Bottom* acceptance, tracking efficiency (*left* scale), and misreconstructed-track rate (*right* scale) in the range $|\eta | < 2.4$ as a function of $p_{\mathrm {T}}$ for positively charged pions, kaons, and protons

The simulated ratio of the data selection efficiency to the DS selection efficiency is shown as a function of the reconstructed track multiplicity in the top panel of Fig. 1. The ratio is used to correct the measured events. The results are also corrected for the fraction of DS events without a reconstructed track. This fraction, as given by the simulation, is about 0.1 %.

The extrapolation of particle spectra into the unmeasured $(y,p_{\mathrm {T}})$ regions is model dependent, particularly at low $p_{\mathrm {T}}$. A high-precision measurement therefore requires reliable track reconstruction down to the lowest possible $p_{\mathrm {T}}$. The present analysis extends to $p_{\mathrm {T}} \approx 0.1{\,\text {GeV/}c}$ by exploiting special tracking algorithms [18], used in previous studies [8, 16, 19], to provide high reconstruction efficiency and low background rate. The charged-pion mass was assumed when fitting particle momenta.

The acceptance of the tracker ($C_\mathrm{a}$) is defined as the fraction of primary charged particles leaving at least two hits in the pixel detector. It is flat in the region $-2 < \eta < 2$ and $p_{\mathrm {T}} > 0.4{\,\text {GeV/}c}$, and its value is 96–98 % (Fig. 1, bottom panel). The loss of acceptance at $p_{\mathrm {T}} < 0.4{\,\text {GeV/}c}$ is caused by energy loss and multiple scattering of particles, both depending on the particle mass. Likewise, the reconstruction efficiency ($C_\mathrm{e}$) is about 75–85 %, degrading at low $p_{\mathrm {T}}$, also in a mass-dependent way. The misreconstructed-track rate ($C_f$) is very small, reaching 1 % only for $p_{\mathrm {T}} <$ 0.2${\,\text {GeV/}c}$. The probability of reconstructing multiple tracks ($C_\mathrm{m}$) from a single true track is about 0.1 %, mostly due to particles spiralling in the strong magnetic field of the CMS solenoid. The efficiencies and background rates do not depend on the charged-multiplicity of the event. They largely factorize in $\eta$ and $p_{\mathrm {T}}$, but for the final corrections an $(\eta ,p_{\mathrm {T}})$ matrix is used.

The region where pPb collisions occur (beam spot) is measured by reconstructing vertices from many events. Since the bunches are very narrow in the transverse direction, the $xy$ location of the interaction vertices is well constrained; conversely, their $z$ coordinates are spread over a relatively long distance and must be determined on an event-by-event basis. The vertex position is determined using reconstructed tracks which have $p_{\mathrm {T}} > 0.1{\,\text {GeV/}c}$ and originate from the vicinity of the beam spot, i.e. their transverse impact parameters $d_\mathrm{T}$ satisfy the condition $d_\mathrm{T} < 3\,\sigma _T$. Here $\sigma _\mathrm{T}$ is the quadratic sum of the uncertainty in the value of $d_\mathrm{T}$ and the root-mean-square of the beam spot distribution in the transverse plane. The agglomerative vertex-reconstruction algorithm [20] was used, with the $z$ coordinates (and their uncertainties) of the tracks at the point of closest approach to the beam axis as input. For single-vertex events, there is no minimum requirement on the number of tracks associated with the vertex, even one-track vertices are allowed. Only tracks associated with a primary vertex are used in the analysis. If multiple vertices are present, the tracks from the highest multiplicity vertex are used. The resultant bias is negligible since the pileup rate is extremely small.

The vertex reconstruction resolution in the $z$ direction is a strong function of the number of reconstructed tracks and it is always smaller than 0.1$\,\text {cm}$. The distribution of the $z$ coordinates of the reconstructed primary vertices is Gaussian, with a standard deviation of 7.1$\,\text {cm}$. The simulated data were reweighted so as to have the same vertex $z$ coordinate distribution as the data.

The hadron spectra were corrected for particles of non-primary origin ($\tau > 10^{-12}\,\text {s}$). The main sources of secondary particles are weakly decaying particles, mostly $\mathrm {K^0_S}$, $\mathrm {\Lambda }$/$\mathrm {\overline{\Lambda }}$, and $\mathrm {\Sigma ^+}$/$\mathrm {\overline{\Sigma }^-}$. While the correction ($C_\mathrm{s}$) is around 1 % for pions, it rises up to 15 % for protons with $p_{\mathrm {T}} \approx 0.2{\,\text {GeV/}c}$. As none of the mentioned weakly decaying particles decay into kaons, the correction for kaons is small. Based on studies comparing reconstructed $\mathrm {K^0_S}$, $\mathrm {\Lambda }$, and $\mathrm {\overline{\Lambda }}$ spectra and predictions from the Hijing event generator, the corrections are reweighted by a $p_{\mathrm {T}}$-dependent factor.

For $p < 0.15{\,\text {GeV/}c}$, electrons can be clearly identified. The overall $\mathrm {e}^\pm$ contamination of the hadron yields is below 0.2 %. Although muons cannot be separated from pions, their fraction is very small, below 0.05 %. Since both contaminations are negligible, no corrections are applied for them.

## Estimation of energy loss rate and yield extraction

In this paper an analytical parametrization [21] has been used to approximate the energy loss of charged particles in the silicon detectors. The method provides the probability density $P(\Delta |\varepsilon , l)$ of energy deposit $\Delta$, if the most probable energy loss rate $\varepsilon$ at a reference path-length $l_0 = 450\,\upmu \text {m}$ and the path-length $l$ are known. It was used in conjunction with a maximum likelihood method, for the estimate of $\varepsilon$.

For pixel clusters, the energy deposits were calculated as the sum of individual pixel deposits. In the case of strips, the energy deposits were corrected for capacitive coupling and cross-talk between neighboring strips. The readout threshold, the coupling parameter, and the standard deviation of the Gaussian noise for strips were determined from data, using tracks with close-to-normal incidence.

For an accurate determination of $\varepsilon$, the response of all readout chips was calibrated with multiplicative gain correction factors. The measured energy deposit spectra were compared to the energy loss parametrization and hit-level corrections (affine transformation of energy deposits using scale factors and shifts) were introduced. The corrections were applied to individual hits during the determination of the $\ln \varepsilon$ fit templates (described below).

The best value of $\varepsilon$ for each track was calculated with the corrected energy deposits by minimizing the joint energy deposit negative log-likelihood of all hits on the trajectory (index $i$), $\chi ^2 = -2 \sum _i \ln P(\Delta _i|\varepsilon ,l_i)$. Hits with incompatible energy deposits (contributing more than 12 to the joint $\chi ^2$) were excluded. At most one hit was removed; this affected about 1.5 % of the tracks.

**Fig. 2:**
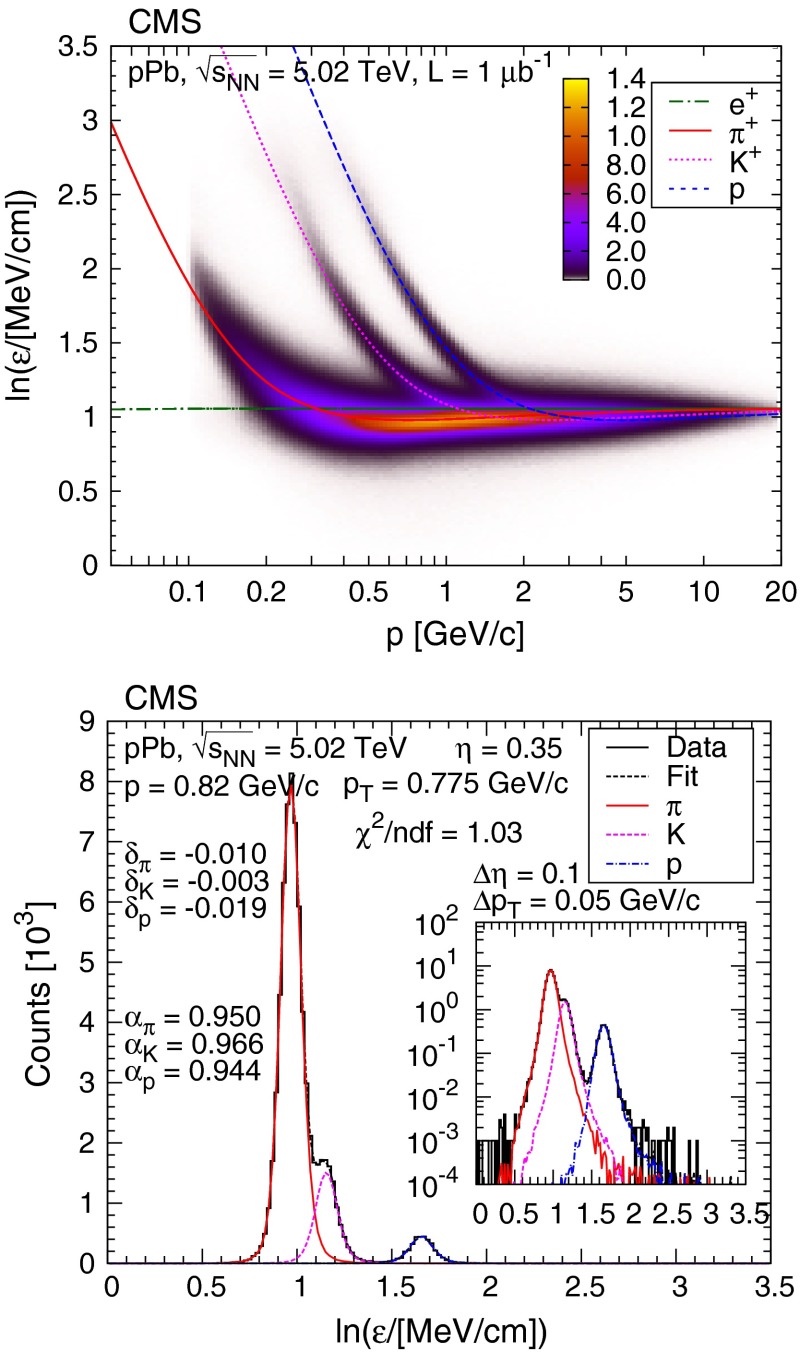
*Top* distribution of $\ln \varepsilon$ as a function of total momentum $p$, for positively charged particles ($\varepsilon$ is the most probable energy loss rate at a reference path length $l_0 = 450\,\upmu \text {m}$). The $z$ scale is shown in arbitrary units and is linear. The curves show the expected $\ln \varepsilon$ for electrons, pions, kaons, and protons (Eq. (30.11) in Ref. [22]). *Bottom* example $\ln \varepsilon$ distribution at $\eta = 0.35$ and $p_{\mathrm {T}} = 0.775{\,\text {GeV/}c}$, with bin widths $\Delta \eta = 0.1$ and $\Delta p_{\mathrm {T}} = 0.05 {\,\text {GeV/}c}$. Scale factors ($\alpha$) and shifts ($\delta$) are indicated (see text). The *inset* shows the distribution with logarithmic vertical scale

Distributions of $\ln \varepsilon$ as a function of total momentum $p$ for positive particles are plotted in the top panel of Fig. 2 and compared to the predictions of the energy loss method [21] for electrons, pions, kaons, and protons. The remaining deviations were taken into account by means of track-level corrections mentioned above (affine transformation of templates using scale factors and shifts, $\ln \varepsilon \rightarrow \alpha \ln \varepsilon + \delta$).

Low-momentum particles can be identified unambiguously and can therefore be counted. Conversely, at high momentum, the $\ln \varepsilon$ bands overlap (above about 0.5${\,\text {GeV/}c}$ for pions and kaons and 1.2${\,\text {GeV/}c}$ for protons); the particle yields therefore need to be determined by means of a series of template fits in $\ln \varepsilon$, in bins of $\eta$ and $p_{\mathrm {T}}$ (Fig. 2, bottom panel). Finally, fit templates, giving the expected $\ln \varepsilon$ distributions for all particle species (electrons, pions, kaons, and protons), were built from tracks. All kinematical parameters and hit-related observables were kept, but the energy deposits were regenerated by sampling from the analytical parametrization. For a less biased determination of track-level residual corrections, enhanced samples of each particle type were employed. These were used for setting starting values of the fits. For electrons and positrons, photon conversions in the beam-pipe and innermost first pixel layer were used. For high-purity ${\pi }$ and enhanced $\mathrm {p}$ samples, weakly decaying hadrons were selected ($\mathrm {K^0_S}$, $\mathrm {\Lambda }$/$\mathrm {\overline{\Lambda }}$). The relations and constraints described in Ref. [8] were also exploited, this way better constraining the parameters of the fits: fitting the $\ln \varepsilon$ distributions in number of hits (${n_\mathrm{hits}}$) and track-fit $\chi ^2/\hbox {ndf}$ slices simultaneously; fixing the distribution ${n_\mathrm{hits}}$ of particle species, relative to each other; using the expected continuity for refinement of track-level residual corrections, in neighboring $(\eta ,p_{\mathrm {T}})$ bins; using the expected convergence for track-level residual corrections, as the $\ln \varepsilon$ values of two particle species approach each other.

The results of the (iterative) $\ln \varepsilon$ fits are the yields for each particle species and charge in bins of $(\eta ,p_{\mathrm {T}})$ or $(y,p_{\mathrm {T}})$, both inclusive and divided into classes of reconstructed primary charged-track multiplicity. In the end, the histogram fit $\chi ^2/\hbox {ndf}$ values were usually close to unity. Although pion and kaon yields could not be determined for $p > 1.30{\,\text {GeV/}c}$, their sum was measured. This information is an important constraint when fitting the $p_{\mathrm {T}}$ spectra.

The statistical uncertainties for the extracted yields are given by the fits. The observed local variations of parameters in the $(\eta ,p_{\mathrm {T}})$ plane for track-level corrections cannot be attributed to statistical fluctuations and indicate that the average systematic uncertainties in the scale factors and shifts are about $10^{-2}$ and $2 \times 10^{-3}$, respectively. These scale factors and shifts agree with those seen in the high-purity samples to well within a factor of two. The systematic uncertainties in the yields in each bin were obtained by refitting the histograms with the parameters changed by these amounts.

## Corrections and systematic uncertainties

The measured yields in each $(\eta ,p_{\mathrm {T}})$ bin, $\Delta N_\mathrm{measured}$, were first corrected for the misreconstructed-track rate ($C_\mathrm{f}$) and the fraction of secondary particles ($C_\mathrm{s}$):

1 $$\begin{aligned} \Delta N' = \Delta N_\mathrm{measured} \cdot (1 - C_\mathrm{f}) \cdot (1 - C_\mathrm{s}). \end{aligned}$$

The distributions were then unfolded to take into account the finite $\eta$ and $p_{\mathrm {T}}$ resolutions. The $\eta$ distribution of the tracks is almost flat and the $\eta$ resolution is very good. Conversely, the $p_{\mathrm {T}}$ distribution is steep in the low-momentum region and separate corrections in each $\eta$ bin were necessary. An unfolding procedure with linear regularization [23] was used, based on response matrices obtained from MC samples for each particle species.

The corrected yields were obtained by applying corrections for acceptance ($C_\mathrm{a}$), efficiency ($C_\mathrm{e}$), and multiple track reconstruction rate ($C_\mathrm{m}$):

2 $$\begin{aligned} \frac{1}{N_\mathrm{ev}} \frac{\text {d}^2 N}{\text {d}\eta \, \text {d}p_{\mathrm {T}}}_\mathrm{corrected} = \frac{1}{C_\mathrm{a} \cdot C_\mathrm{e} \cdot (1 + C_\mathrm{m})} \frac{\Delta N'}{N_\mathrm{ev} \Delta \eta \Delta p_{\mathrm {T}}}, \end{aligned}$$

where $N_\mathrm{ev}$ is the corrected number of DS events (Fig. 1). Bins with acceptance smaller than 50 %, efficiency smaller than 50 %, multiple-track rate greater than 10 %, or containing less than 80 tracks were not used.

Finally, the differential yields $\text {d}^2N/\text {d}\eta \,\text {d}p_{\mathrm {T}}$ were transformed to invariant yields $\text {d}^2N/\text {d}y\,\text {d}p_{\mathrm {T}}$ by multiplying with the Jacobian $E/p$ and the $(\eta ,p_{\mathrm {T}})$ bins were mapped into a $(y,p_{\mathrm {T}})$ grid. As expected, there is a small (5–10 %) $y$ dependence in the narrow region considered ($|y |<1$), depending on event multiplicity. The yields as a function of $p_{\mathrm {T}}$ were obtained by averaging over rapidity.

**Fig. 3:**
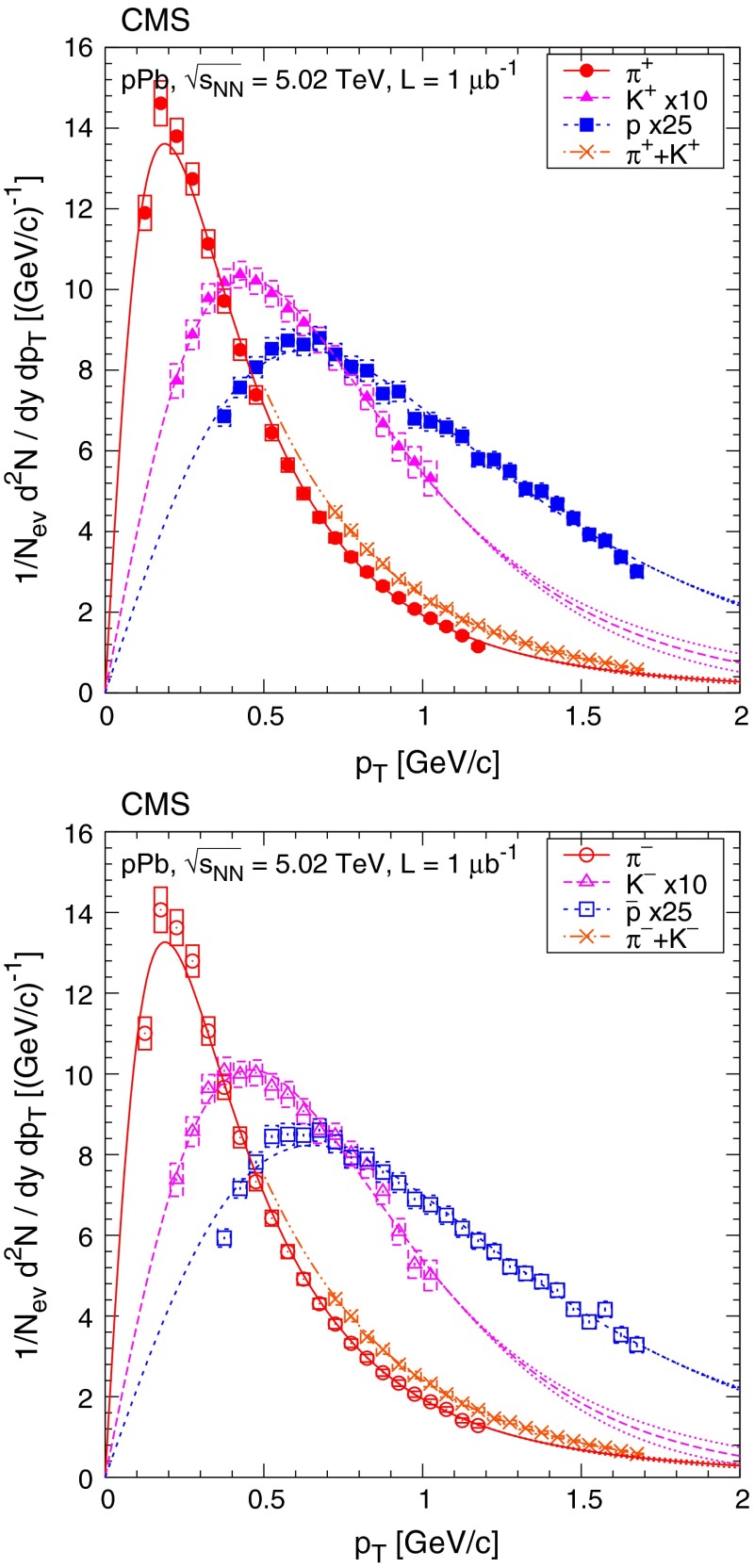
Transverse momentum distributions of identified charged hadrons (pions, kaons, protons, sum of pions and kaons) in the range $|y |<1$, for positively (*top*) and negatively (*bottom*) charged particles. Kaon and proton distributions are scaled as shown in the legends. Fits to Eqs. (3) and (5) are superimposed. *Error bars* indicate the uncorrelated statistical uncertainties, while *boxes* show the uncorrelated systematic uncertainties. The fully correlated normalization uncertainty (not shown) is 3.0 %. *Dotted lines* illustrate the effect of varying the $1/n$ value of the Tsallis–Pareto function by $\pm 0.1$ above the highest measured $p_{\mathrm {T}}$ point

The systematic uncertainties are very similar to those in Ref. [8] and are summarized in Table 1. The uncertainties of the corrections related to the event selection and pileup are fully or mostly correlated and were treated as normalization uncertainties: 3.0 % uncertainty on the yields and 1.0 % on the average $p_{\mathrm {T}}$. In order to study the influence of the high $p_{\mathrm {T}}$ extrapolation on $\langle \text {d}N/\text {d}y\rangle$ and $\langle p_{\mathrm {T}} \rangle$, the $1/n$ parameter of the fitted Tsallis–Pareto function (Sect. 5) was varied. While keeping the function in the measured range, $1/n$ was increased and decreased by $\pm 0.1$ above the highest $p_{\mathrm {T}}$ measured point, ensuring that the two function pieces are continuous both in value and derivative. The choice of the magnitude for the variation was motivated by the fitted $1/n$ values and their distance from a Boltzmann distribution. (The resulting functions are plotted in Fig. 3 as dotted lines.) The high $p_{\mathrm {T}}$ extrapolation introduces sizeable systematic uncertainties, 4–6 % for $\langle \text {d}N/\text {d}y\rangle$, and 9–15 % for $\langle p_{\mathrm {T}} \rangle$ in case of the DS selection.

**Table 1:**
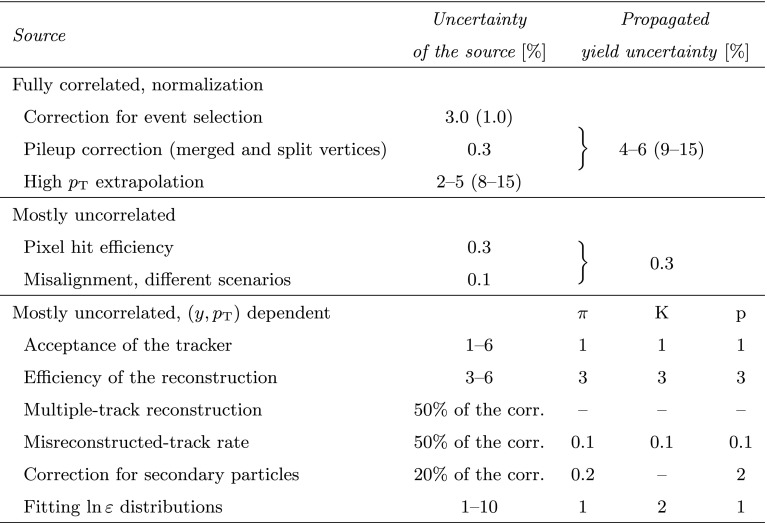
Summary of the systematic uncertainties affecting the $p_{\mathrm {T}}$ spectra. Values in parentheses indicate uncertainties in the $\langle p_{\mathrm {T}} \rangle$ measurement. The systematic uncertainty related to the low $p_{\mathrm {T}}$ extrapolation is small compared to the contributions from other sources and therefore not included in the combined systematic uncertainty of the measurement. Representative, particle-specific uncertainties (${\pi }$, $\mathrm {K}$, $\mathrm {p}$) are given for $p_{\mathrm {T}} =0.6{\,\text {GeV/}c}$ in the third group of systematic uncertainties

The tracker acceptance and the track reconstruction efficiency generally have small uncertainties (1 and 3 %, respectively), but change rapidly at very low $p_{\mathrm {T}}$ (bottom panel of Fig. 1), leading to a 6 % uncertainty on the yields in that range. For the multiple-track and misreconstructed-track rate corrections, the uncertainty is assumed to be 50 % of the correction, while for the case of the correction for secondary particles it was estimated to be 20 %. These mostly uncorrelated uncertainties are due to the imperfect modeling of the detector: regions with mismodeled efficiency in the tracker, alignment uncertainties, and channel-by-channel varying hit efficiency. These circumstances can change frequently in momentum space, so can be treated as uncorrelated.

The systematic uncertainties originating from the unfolding procedure were studied. Since the $p_{\mathrm {T}}$ response matrices are close to diagonal, the unfolding of $p_{\mathrm {T}}$ distributions did not introduce substantial systematics. At the same time the inherited uncertainties were properly propagated. The introduced correlations between neighboring $p_{\mathrm {T}}$ bins were neglected, hence statistical uncertainties were regarded as uncorrelated while systematic uncertainties were expected to be locally correlated in $p_{\mathrm {T}}$. The systematic uncertainty of the fitted yields is in the range 1–10 % depending mostly on total momentum.

## Results

In previously published measurements of unidentified and identified particle spectra [16, 24], the following form of the Tsallis–Pareto-type distribution [25, 26] was fitted to the data:

3 $$\begin{aligned} \frac{\text {d}^2 N}{\text {d}y \, \text {d}p_{\mathrm {T}}} = \frac{\text {d}N}{\text {d}y} \cdot C \cdot p_{\mathrm {T}} \left[ 1 + \frac{m_{\mathrm {T}}- m}{nT} \right] ^{-n}, \end{aligned}$$

where

4 $$\begin{aligned} C = \frac{(n-1)(n-2)}{nT[nT + (n-2) m]} \end{aligned}$$

and $m_{\mathrm {T}} = \sqrt{m^2 + p_{\mathrm {T}} ^2}$ (factors of $c$ are omitted from the preceding formulae). The free parameters are the integrated yield $\text {d}N/\text {d}y$, the exponent $n$, and parameter $T$. The above formula is useful for extrapolating the spectra to zero $p_{\mathrm {T}}$ and very high $p_{\mathrm {T}}$ and for extracting $\langle p_{\mathrm {T}} \rangle$ and $\text {d}N/\text {d}y$. Its validity for different multiplicity bins was cross-checked by fitting MC spectra in the $p_{\mathrm {T}}$ ranges where there are data points, and verifying that the fitted values of $\langle p_{\mathrm {T}} \rangle$ and $\text {d}N/\text {d}y$ were consistent with the generated values. Nevertheless, for a more robust estimation of both quantities ($\langle p_{\mathrm {T}} \rangle$ and $\langle \text {d}N/\text {d}y \rangle$), the data points and their uncertainties were used in the measured range and the fitted functions only for the extrapolation in the unmeasured regions. According to some models of particle production based on non-extensive thermodynamics [26], the parameter $T$ is connected with the average particle energy, while $n$ characterizes the “non-extensivity” of the process, i.e. the departure of the spectra from a Boltzmann distribution ($n = \infty$).

As discussed earlier, pions and kaons cannot be unambiguously distinguished at higher momenta. Because of this, the pion-only, the kaon-only, and the joint pion and kaon $\text {d}^2N/\text {d}y \, \text {d}p_{\mathrm {T}}$ distributions were fitted for $|y | < 1$ and $p < 1.20{\,\text {GeV/}c}$, $|y | < 1$ and $p < 1.05{\,\text {GeV/}c}$, and $|\eta | <1$ and $1.05 < p < 1.7{\,\text {GeV/}c}$, respectively. Since the ratio $p/E$ for the pions (which are more abundant than kaons) at these momenta can be approximated by $p_{\mathrm {T}}/m_{\mathrm {T}}$ at $\eta \approx 0$, Eq. (3) becomes:

5 $$\begin{aligned} \frac{\text {d}^2 N}{\text {d}\eta \, \text {d}p_{\mathrm {T}}} \approx \frac{\text {d}N}{\text {d}y} \cdot C \cdot \frac{p_{\mathrm {T}} ^2}{m_{\mathrm {T}}} \left( 1 + \frac{m_{\mathrm {T}}-m}{nT} \right) ^{-n}. \end{aligned}$$

The approximate fractions of particles outside the measured $p_{\mathrm {T}}$ range depend on track multiplicity; they are 15–30 % for pions, 40–50 % for kaons, and 20–35 % for protons. The average transverse momentum $\langle p_{\mathrm {T}} \rangle$ and its uncertainty were obtained using data points in the measured range complemented by numerical integration of Eq. (3) with the fitted parameters in the unmeasured regions, under the assumption that the particle yield distributions follow the Tsallis–Pareto function in the low-$p_{\mathrm {T}}$ and high-$p_{\mathrm {T}}$ regions.

The results discussed in the following are for laboratory rapidity $|y | < 1$. In all cases, error bars indicate the uncorrelated statistical uncertainties, while boxes show the uncorrelated systematic uncertainties. The fully correlated normalization uncertainty is not shown. For the $p_{\mathrm {T}}$ spectra, the average transverse momentum, and the ratio of particle yields, the data are compared to Ampt 1.26/2.26 [13], Epos Lhc [14, 15], and Hijing 2.1 [11] MC event generators. Numerical results corresponding to the plotted spectra, fit results, as well as their statistical and systematic uncertainties are given in Ref. [27].

### Inclusive measurements

**Fig. 4:**
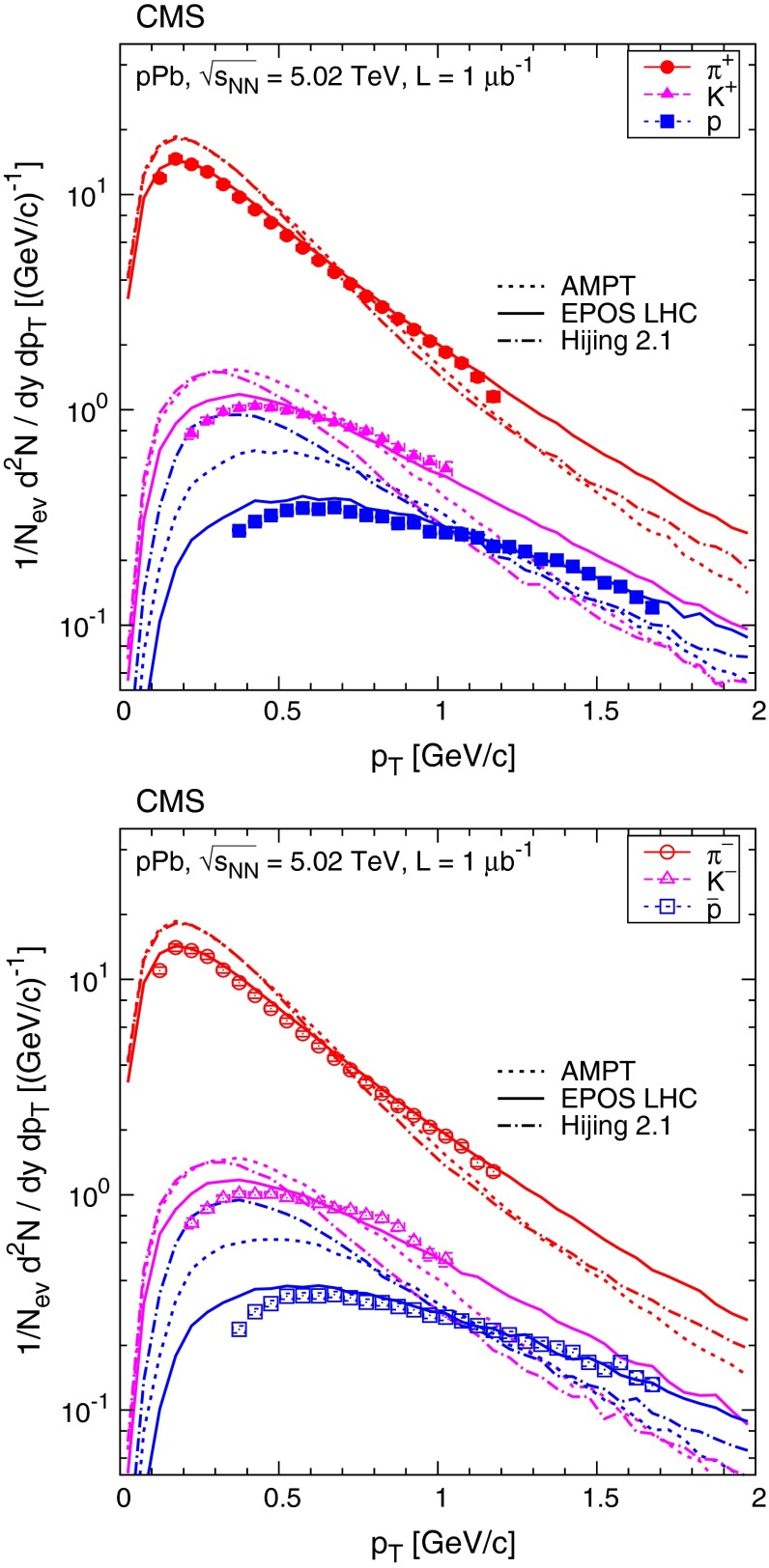
Transverse momentum distributions of identified charged hadrons (pions, kaons, protons) in the range $|y |<1$, for positively (*top*) and negatively (*bottom*) charged particles. Measured values (same as in Fig. 3) are plotted together with predictions from Ampt, Epos Lhc, and Hijing. *Error bars* indicate the uncorrelated statistical uncertainties, while *boxes* show the uncorrelated systematic uncertainties. The fully correlated normalization uncertainty (not shown) is 3.0 %

The transverse momentum distributions of positively and negatively charged hadrons (pions, kaons, protons) are shown in Fig. 3, along with the results of the fits to the Tsallis–Pareto parametrization (Eqs. (3) and (5)). The fits are of good quality with $\chi ^2/\hbox {ndf}$ values in the range 0.4–2.8 (Table 2). Figure 4 presents the data compared to the Ampt, Epos Lhc, and Hijing predictions. Epos Lhc gives a good description, while other generators predict steeper $p_{\mathrm {T}}$ distributions than found in data.

**Table 2:** Fit results ($\text {d}N/\text {d}y$, $1/n$, and $T$) and goodness-of-fit values for the DS selection shown together with calculated averages ($\langle \text {d}N/\text {d}y\rangle$, $\langle p_{\mathrm {T}} \rangle$) for charged pions, kaons, and protons. The systematic uncertainty related to the low $p_{\mathrm {T}}$ extrapolation is small compared to the contributions from other sources and therefore not included in the combined systematic uncertainty of the measurement. Combined uncertainties are given

Particle	$\text {d}N/\text {d}y$	$1/n$	$T$ (${\text {GeV/}c} {}$)	$\chi ^2/\hbox {ndf}$	$\langle \text {d}N/\text {d}y \rangle$	$\langle p_{\mathrm {T}} \rangle$ (${\text {GeV/}c} {}$)
$\mathrm {\pi ^+}$	8.074 $\pm$ 0.081	0.190 $\pm$ 0.007	0.131 $\pm$ 0.003	0.88	8.064 $\pm$ 0.190	0.547 $\pm$ 0.078
$\mathrm {\pi ^-}$	7.971 $\pm$ 0.079	0.195 $\pm$ 0.007	0.131 $\pm$ 0.003	1.05	7.966 $\pm$ 0.196	0.559 $\pm$ 0.083
$\mathrm {K^+}$	1.071 $\pm$ 0.068	0.092 $\pm$ 0.066	0.278 $\pm$ 0.022	0.42	1.040 $\pm$ 0.053	0.790 $\pm$ 0.104
$\mathrm {K^-}$	0.984 $\pm$ 0.047	$-$0.008 $\pm$ 0.067	0.316 $\pm$ 0.024	2.82	0.990 $\pm$ 0.037	0.744 $\pm$ 0.061
$\mathrm {p}$	0.510 $\pm$ 0.018	0.151 $\pm$ 0.036	0.325 $\pm$ 0.016	0.81	0.510 $\pm$ 0.024	1.243 $\pm$ 0.183
$\mathrm {\overline{p}}$	0.494 $\pm$ 0.017	0.123 $\pm$ 0.038	0.349 $\pm$ 0.017	1.32	0.495 $\pm$ 0.022	1.215 $\pm$ 0.165

**Fig. 5:**
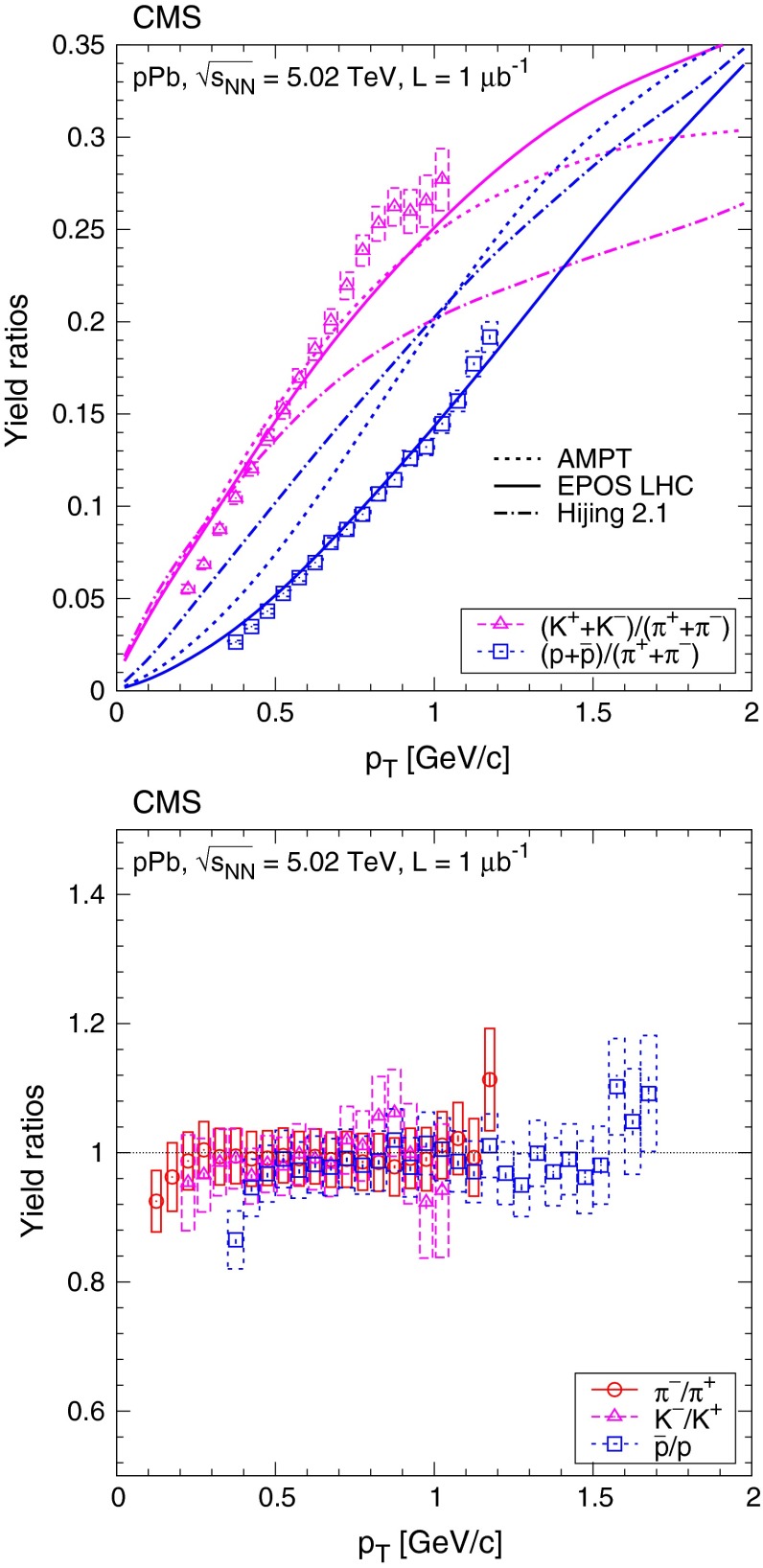
Ratios of particle yields as a function of transverse momentum. $\mathrm {K}$/${\pi }$ and $\mathrm {p}$/${\pi }$ values are shown in the *top* panel, and opposite-charge ratios are plotted in the *bottom* panel. *Error bars* indicate the uncorrelated statistical uncertainties, while *boxes* show the uncorrelated systematic uncertainties. In the *top* panel, *curves* indicate predictions from Ampt, Epos Lhc, and Hijing

Ratios of particle yields as a function of the transverse momentum are plotted in Fig. 5. While the $\mathrm {K}/{\pi }$ ratios are well described by the Ampt simulation, only Epos Lhc is able to predict both $\mathrm {K}/{\pi }$ and $\mathrm {p}/{\pi }$ ratios. The ratios of the yields for oppositely charged particles are close to one, as expected for LHC energies at midrapidity.

### Multiplicity dependent measurements

A study of the dependence on track multiplicity is motivated partly by the intriguing hadron correlations measured in pp and pPb collisions at high track multiplicities [28–31], suggesting possible collective effects in “central” pp and pPb collisions at the LHC. At the same time, it was seen that in pp collisions the characteristics of particle production ($\langle p_{\mathrm {T}} \rangle$, ratios) at LHC energies are strongly correlated with event particle multiplicity rather than with the center-of-mass energy of the collision [8]. The strong dependence on multiplicity (or centrality) was also seen in dAu collisions at RHIC [6, 7]. In addition, the multiplicity dependence of particle yield ratios is sensitive to various final-state effects (hadronization, color reconnection, collective flow) implemented in MC models used in collider and cosmic-ray physics [32].

**Fig. 6:**
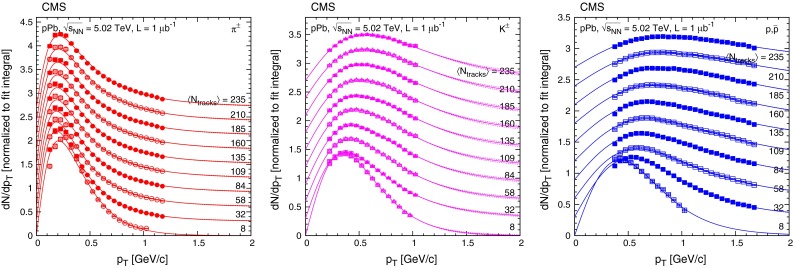
Transverse momentum distributions of charged pions, kaons, and protons, normalized such that the fit integral is unity, in every second multiplicity class ($\langle N_\mathrm{tracks} \rangle$ values are indicated) in the range $|y |<1$, fitted with the Tsallis–Pareto parametrization (*solid lines*). For better visibility, the result for any given $\langle N_\mathrm{tracks} \rangle$ bin is shifted by 0.3 units with respect to the adjacent bins. *Error bars* indicate the uncorrelated statistical uncertainties, while *boxes* show the uncorrelated systematic uncertainties. *Dotted lines* illustrate the effect of varying the $1/n$ value of the Tsallis–Pareto function by $\pm 0.1$ above the highest measured $p_{\mathrm {T}}$ point

**Fig. 7:**
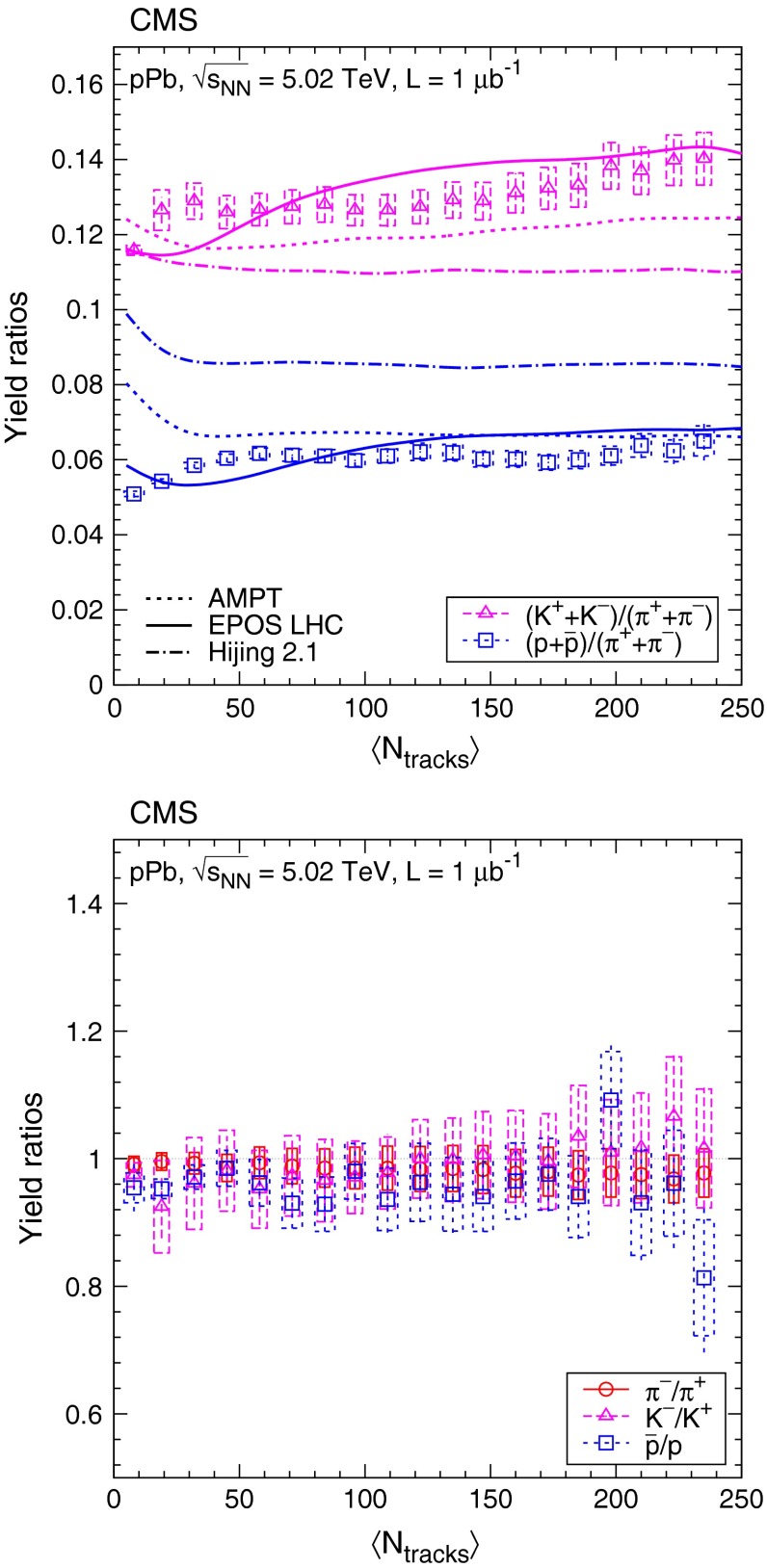
Ratios of particle yields in the range $|y |<1$ as a function of the corrected track multiplicity for $|\eta |<2.4$. $\mathrm {K}$/${\pi }$ and $\mathrm {p}$/${\pi }$ values are shown in the *top* panel, and opposite-charge ratios are plotted in the *bottom* panel. *Error bars* indicate the uncorrelated combined uncertainties, while *boxes* show the uncorrelated systematic uncertainties. In the *top* panel, curves indicate predictions from Ampt, Epos Lhc, and Hijing

**Fig. 8:**
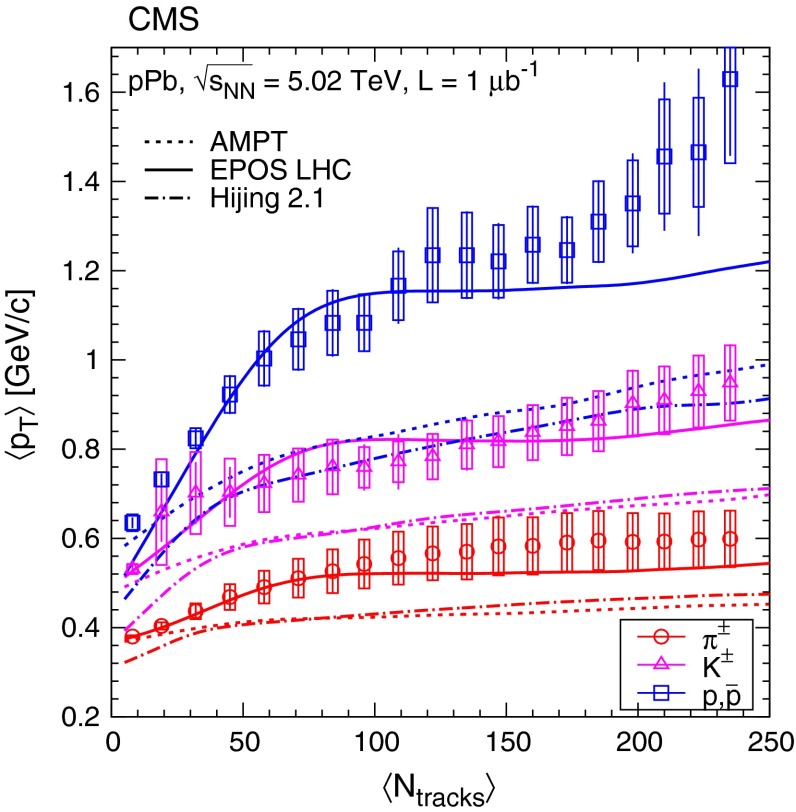
Average transverse momentum of identified charged hadrons (pions, kaons, protons) in the range $|y |<1$, as a function of the corrected track multiplicity for $|\eta |<2.4$, computed assuming a Tsallis–Pareto distribution in the unmeasured range. *Error bars* indicate the uncorrelated combined uncertainties, while boxes show the uncorrelated systematic uncertainties. The fully correlated normalization uncertainty (not shown) is 1.0 %. *Curves* indicate predictions from Ampt, Epos Lhc, and Hijing

**Fig. 9:**
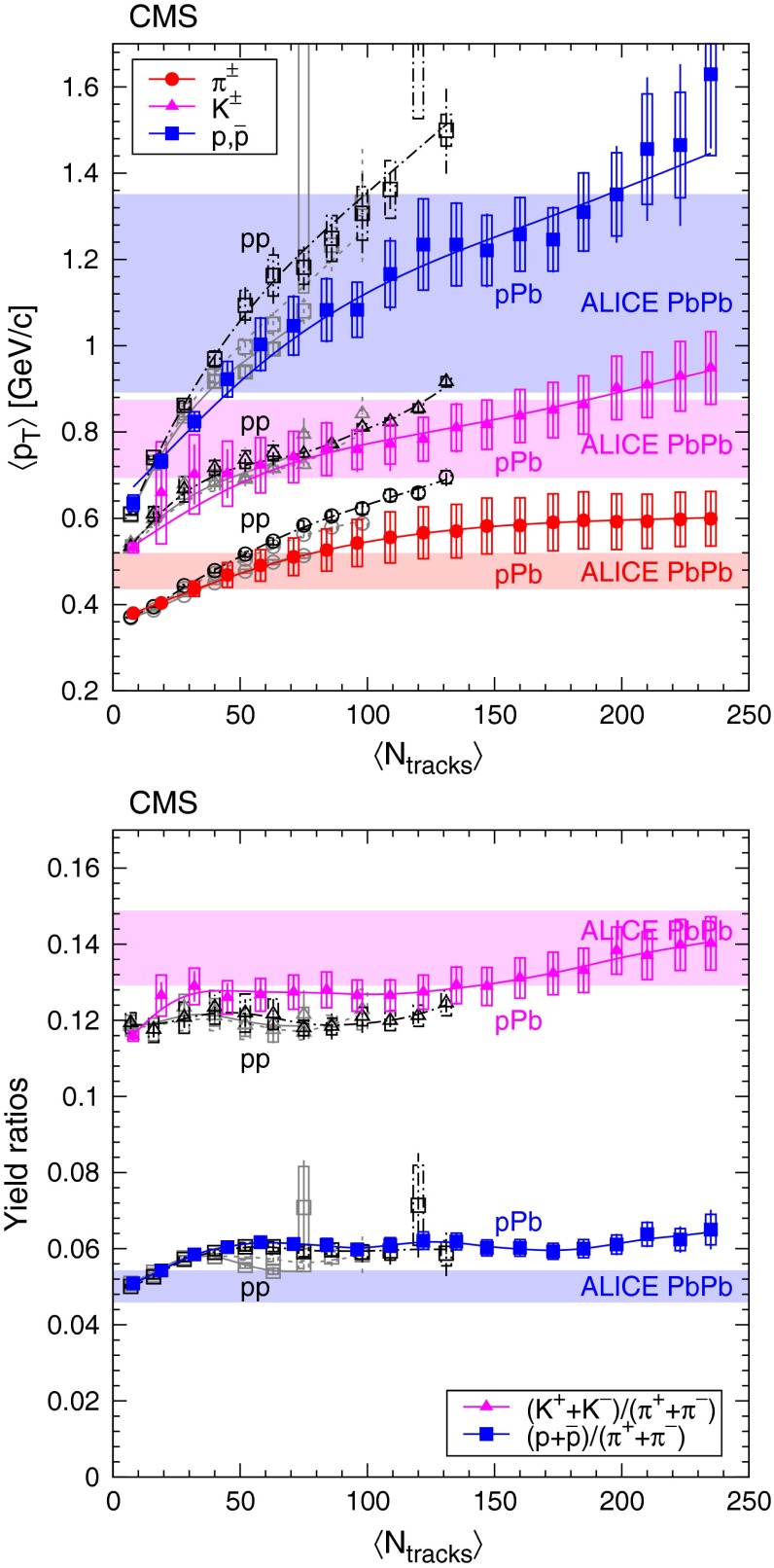
Average transverse momentum of identified charged hadrons (pions, kaons, protons; *top* panel) and ratios of particle yields (*bottom* panel) in the range $|y |<1$ as a function of the corrected track multiplicity for $|\eta |<2.4$, for pp collisions (*open symbols*) at several energies [8], and for pPb collisions (*filled symbols*) at $\sqrt{s_{NN}} =$ 5.02$\,\text {TeV}$. Both $\langle p_{\mathrm {T}} \rangle$ and yield ratios were computed assuming a Tsallis–Pareto distribution in the unmeasured range. *Error bars* indicate the uncorrelated combined uncertainties, while *boxes* show the uncorrelated systematic uncertainties. For $\langle p_{\mathrm {T}} \rangle$ the fully correlated normalization uncertainty (not shown) is 1.0 %. In both plots, lines are drawn to guide the eye (*gray solid* pp 0.9$\,\text {TeV}$, *gray dotted* pp 2.76$\,\text {TeV}$, *black dash-dotted* pp 7$\,\text {TeV}$, *colored solid* pPb 5.02$\,\text {TeV}$). The ranges of $\langle p_{\mathrm {T}} \rangle$, $\mathrm {K}/{\pi }$ and $\mathrm {p}/{\pi }$ values measured by ALICE in various centrality PbPb collisions (see text) at $\sqrt{s_{NN}} = 2.76\,\text {TeV}$ [33] are indicated with *horizontal bands*

**Fig. 10:**
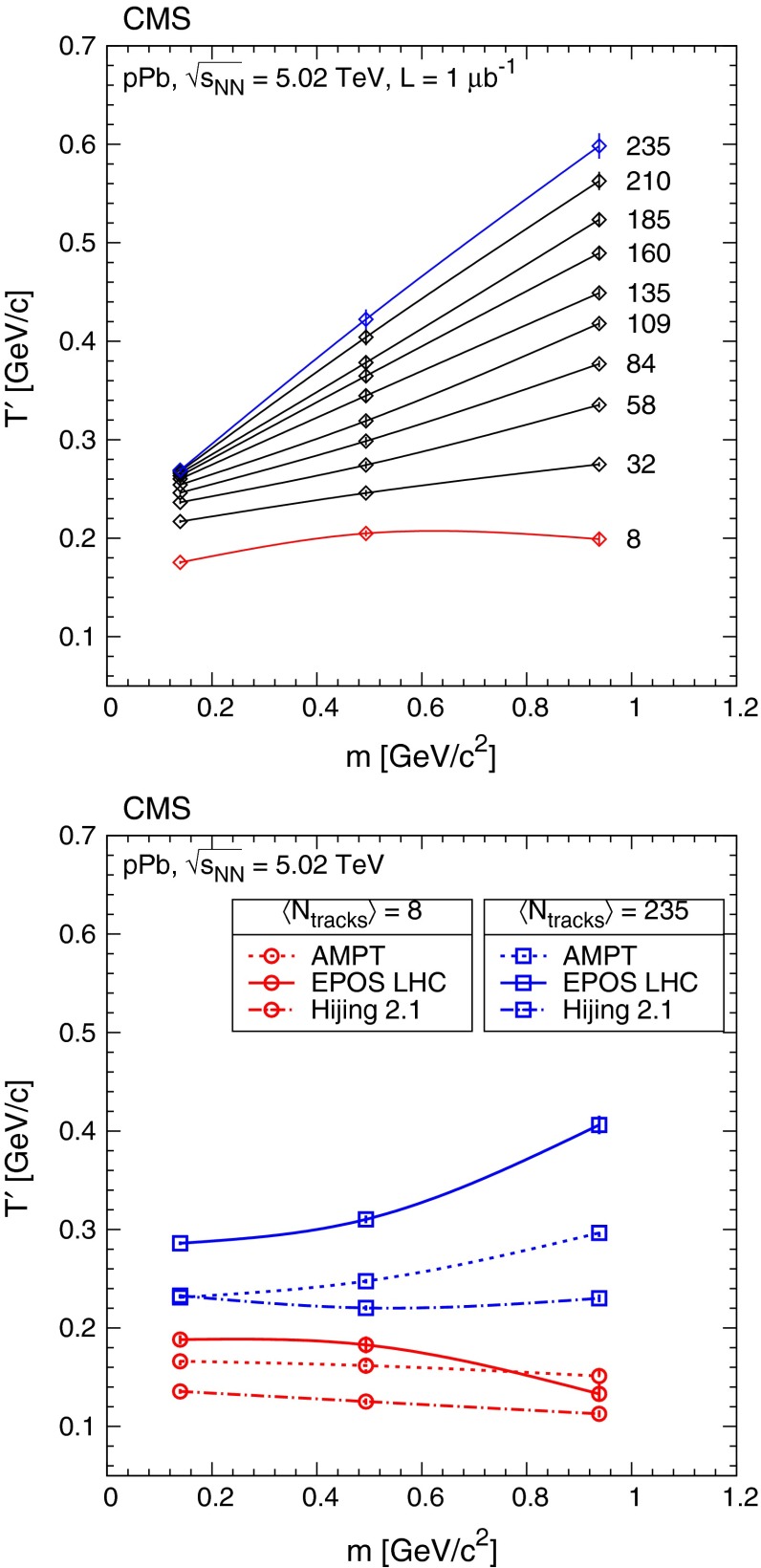
Inverse slope parameters $T'$ from fits of pion, kaon, and proton spectra (both charges) with a form proportional to $p_{\mathrm {T}} \exp (-m_{\mathrm {T}}/T')$. Results for a selection of multiplicity classes, with different $\langle N_\mathrm{tracks} \rangle$ as indicated, are plotted for pPb data (*top*) and for MC event generators Ampt, Epos Lhc, and Hijing (*bottom*). The *curves* are drawn to guide the eye

The event multiplicity $N_\mathrm{rec}$ is obtained from the number of reconstructed tracks with $|\eta |<2.4$, where the tracks are reconstructed using the same algorithm as for the identified charged hadrons [18]. (The multiplicity variable $N_\mathrm{trk}^\mathrm{offline}$, used in Ref. [29], is obtained from a different track reconstruction configuration and a value of $N_\mathrm{trk}^\mathrm{offline} = 110$ corresponds roughly to $N_\mathrm{rec} = 170$.) The event multiplicity was divided into 19 classes, defined in Table 3. To facilitate comparisons with models, the corresponding corrected charged particle multiplicity in the same acceptance of $|\eta |<2.4$ ($N_\mathrm{tracks}$) is also determined. For each multiplicity class, the correction from $N_\mathrm{rec}$ to $N_\mathrm{tracks}$ uses the efficiency estimated with the Hijing simulation in $(\eta ,p_{\mathrm {T}})$ bins. The corrected data are then integrated over $p_{\mathrm {T}}$, down to zero yield at $p_{\mathrm {T}} = 0$ (with a linear extrapolation below $p_{\mathrm {T}} = 0.1 {\,\text {GeV/}c}$). Finally, the integrals for each eta slice are summed. The average corrected charged-particle multiplicity $\langle N_\mathrm{tracks} \rangle$, and also its values with the condition $p_{\mathrm {T}} > 0.4{\,\text {GeV/}c}$, are shown in Table 3 for each event multiplicity class. The value of $\langle N_\mathrm{tracks} \rangle$ is used to identify the multiplicity class in Figs. 6, 7, 8, 9, and 10.

**Table 3:** Relationship between the number of reconstructed tracks ($N_\mathrm{rec}$) and the average number of corrected tracks ($\langle N_\mathrm{tracks} \rangle$) in the region $|\eta | < 2.4$, and also with the condition $p_{\mathrm {T}} > 0.4{\,\text {GeV/}c}$ (used in Ref. [29]), in the 19 multiplicity classes considered

$N_\mathrm{rec}$	0–9	10–19	20–29	30–39	40–49	50–59	60–69	70–79	80–89	90–99	100–109	110–119	120–129	130–139	140–149	150–159	160–169	170–179	180–189
$\langle N_\mathrm{tracks} \rangle$	8	19	32	45	58	71	84	96	109	122	135	147	160	173	185	198	210	222	235
$\langle N_\mathrm{tracks} \rangle _{p_{\mathrm {T}} > 0.4{\,\text {GeV/}c}}$	3	8	15	22	29	36	43	50	58	65	73	80	87	95	103	110	117	125	133

Transverse-momentum distributions of identified charged hadrons, normalized such that the fit integral is unity, in selected multiplicity classes for $|y | < 1$ are shown in Fig. 6 for pions, kaons, and protons. The distributions of negatively and positively charged particles have been summed. The distributions are fitted with the Tsallis–Pareto parametrization with $\chi ^2/\hbox {ndf}$ values in the range 0.8–4.0 for pions, 0.1–1.1 for kaons, and 0.1–0.7 for protons. For kaons and protons, the parameter $T$ increases with multiplicity, while for pions $T$ slightly increases and the exponent $n$ slightly decreases with multiplicity (not shown).

The ratios of particle yields are displayed as a function of track multiplicity in Fig. 7. The $\mathrm {K}/{\pi }$ and $\mathrm {p}/{\pi }$ ratios are flat, or slightly rising, as a function of $\langle N_\mathrm{tracks} \rangle$. While none of the models is able to precisely reproduce the track multiplicity dependence, the best and worst matches to the overall scale are given by Epos Lhc and Hijing, respectively. The ratios of yields of oppositely charged particles are independent of $\langle N_\mathrm{tracks} \rangle$ as shown in the bottom panel of Fig. 7. The average transverse momentum $\langle p_{\mathrm {T}} \rangle$ is shown as a function of multiplicity in Fig. 8. As expected from the discrepancies between theory and data shown in Fig. 4, Epos Lhc again gives a reasonable description, while the other event generators presented here underpredict the measured values. For the dependence of $T$ on multiplicity (not shown), the predictions match the pion data well; the kaon and proton values are much higher than in Ampt or Hijing.

### Comparisons to pp and PbPb data

The comparison with pp data taken at various center-of-mass energies (0.9, 2.76, and 7$\,\text {TeV}$) [8] is shown in Fig. 9, where the dependence of $\langle p_{\mathrm {T}} \rangle$ and the particle yield ratios ($\mathrm {K}/{\pi }$ and $\mathrm {p}/{\pi }$) on the track multiplicity is shown. The plots also display the ranges of these values measured by ALICE in PbPb collisions at $\sqrt{s_{NN}} =$ 2.76$\,\text {TeV}$ for centralities from peripheral (80–90 % of the inelastic cross-section) to central (0–5 %) [33]. These ALICE PbPb data cover a much wider range of $N_\mathrm{tracks}$ than is shown in the plot. Although PbPb data are not available at $\sqrt{s_{NN}} = 5.02\,\text {TeV}$ for comparison, the evolution of event characteristics from RHIC ($\sqrt{s_{NN}} = 0.2\,\text {TeV}$, [3, 4, 6]) to LHC energies [33] suggests that yield ratios should remain similar, while $\langle p_{\mathrm {T}} \rangle$ values will increase by about 5 % when going from $\sqrt{s_{NN}} =$ 2.76$\,\text {TeV}$ to 5.02$\,\text {TeV}$.

For low track multiplicity ($N_\mathrm{tracks} \lesssim 40$), pPb collisions behave very similarly to pp collisions, while at higher multiplicities ($N_\mathrm{tracks} \gtrsim 50$) the $\langle p_{\mathrm {T}} \rangle$ is lower for pPb than in pp. The first observation can be explained since low-multiplicity events are peripheral pPb collisions in which only a few proton–nucleon collisions are present. Events with more particles are indicative of collisions in which the projectile proton strikes the thick disk of the lead nucleus. Interestingly, the pPb curves (Fig. 9, top panel) can be reasonably approximated by taking the pp values and multiplying their $N_\mathrm{tracks}$ coordinate by a factor of 1.8, for all particle types. In other words, a pPb collision with a given $N_\mathrm{tracks}$ is similar to a pp collision with $0.55 \times N_\mathrm{tracks}$ for produced charged particles in the $|\eta | < 2.4$ range. Both the highest-multiplicity pp and pPb interactions yield higher $\langle p_{\mathrm {T}} \rangle$ than seen in central PbPb collisions. While in the PbPb case even the most central collisions possibly contain a mix of soft (lower-$\langle p_{\mathrm {T}} \rangle$) and hard (higher-$\langle p_{\mathrm {T}} \rangle$) nucleon-nucleon interactions, for pp or pPb collisions the most violent interaction or sequence of interactions are selected.

The transverse momentum spectra could also be successfully fitted ($\chi ^2/\hbox {ndf}$ in the range 0.7–1.8) with a functional form proportional to $p_{\mathrm {T}} \exp (-m_{\mathrm {T}}/T')$, where $T'$ is called the inverse slope parameter, motivated by the success of Boltzmann-type distributions in nucleus–nucleus collisions [34]. In the case of pions, the fitted range was restricted to $m_{\mathrm {T}} > 0.4{\,\text {GeV/}c}$ in order to exclude the region where resonance decays would significantly contribute to the measured spectra. The inverse slope parameter as a function of hadron mass is shown in Fig. 10, for a selection of event classes, both for pPb data and for MC event generators (Ampt, Epos Lhc, and Hijing). While the data display a linear dependence on mass with a slope that increases with particle multiplicity, the models predict a flat or slowly rising behavior versus mass and only limited changes with track multiplicity. This is to be compared with pp results [8], where both data and several tunes of the pythia 6 [35] and pythia 8 event generators show features very similar to those in pPb data. A similar trend is also observed in nucleus–nucleus collisions [3, 6], which is attributed to the effect of radial flow velocity boost [1].

**Fig. 11:**
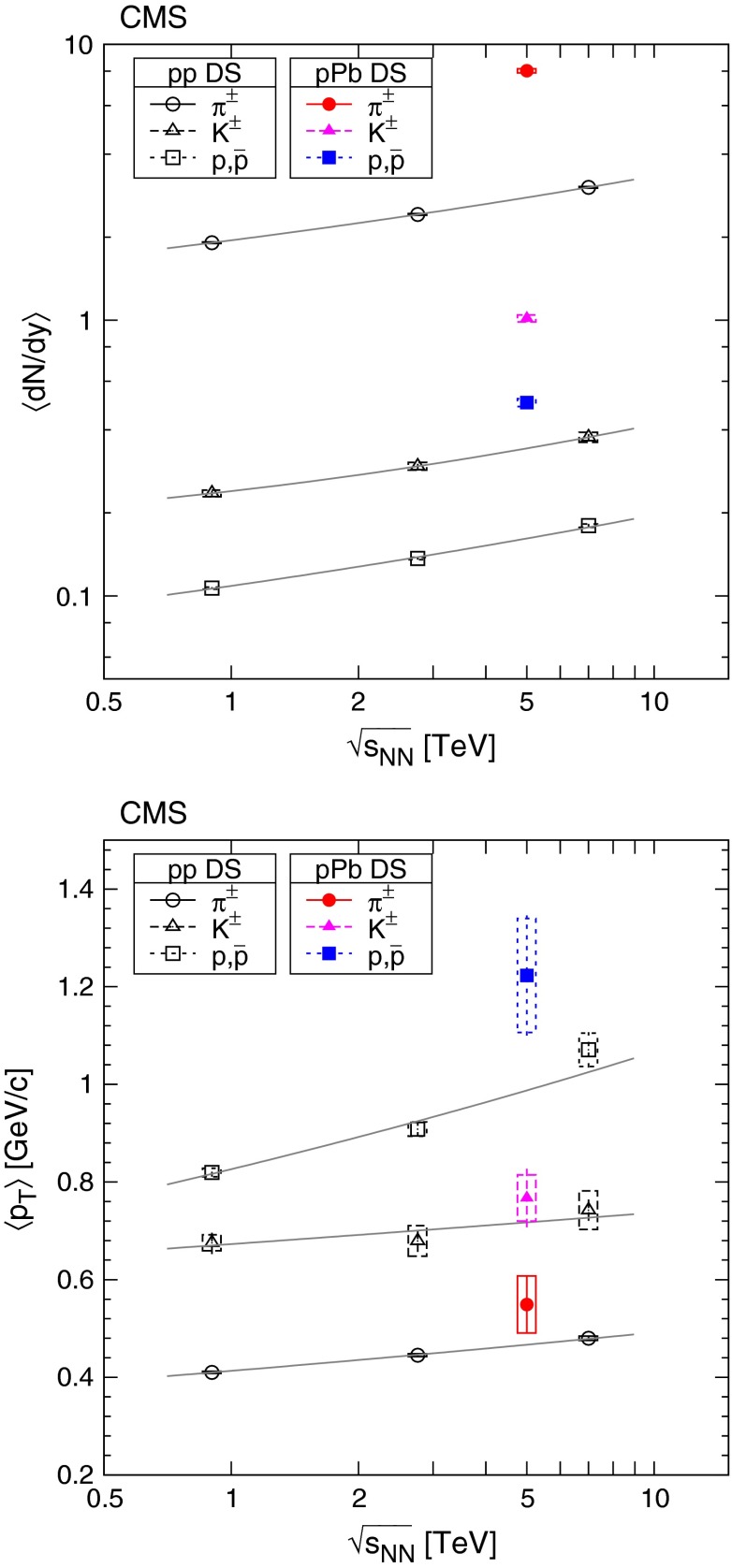
Average rapidity densities $\langle \text {d}N/\text {d}y\rangle$ (*top*) and average transverse momenta $\langle p_{\mathrm {T}} \rangle$ (*bottom*) as a function of center-of-mass energy for pp [8] and pPb collisions, for charge-averaged pions, kaons, and protons. *Error bars* indicate the uncorrelated combined uncertainties, while *boxes* show the uncorrelated systematic uncertainties. The *curves* show parabolic (for $\langle \text {d}N/\text {d}y\rangle$) or linear (for $\langle p_{\mathrm {T}} \rangle$) interpolation on a log-log scale. The pp and pPb data are for laboratory rapidity $|y |<1$, which is the same as the center-of-mass rapidity only for the pp data

Average rapidity densities $\langle \text {d}N/\text {d}y\rangle$ and average transverse momenta $\langle p_{\mathrm {T}} \rangle$ of charge-averaged pions, kaons, and protons as a function of center-of-mass energy are shown in Fig. 11 for pp and pPb collisions, both corrected to the DS selection. To allow comparison at the pPb energy, a parabolic (linear) interpolation of the pp collision values at $\sqrt{s}= 0.9$, 2.76, and 7$\,\text {TeV}$ is shown for $\text {d}N/\text {d}y$ $(\langle p_{\mathrm {T}} \rangle )$. The rapidity densities are generally about three times greater than in pp interactions at the same energy, while the average transverse momentum increases by about 20, 10, and 30 % for pions, kaons, and protons, respectively. The factor of three difference in the yields for pPb as compared to pp can be compared with the estimated number of projectile collisions $N_\mathrm{coll}/2 = 3.5 \pm 0.3$ or with the number of nucleons participating in the collision $N_\mathrm{part}/2 = 4.0 \pm 0.3$, based on the ratio of preliminary pPb and pp cross-section measurements, that have proven to be good scaling variables in proton–nucleus collisions at lower energies [36].

## Conclusions

Measurements of identified charged hadron spectra produced in pPb collisions at $\sqrt{s_{NN}} =5.02\,\text {TeV}$ have been presented, normalized to events with simultaneous hadronic activity at pseudorapidities $-5 < \eta < -3$ and $3 < \eta < 5$. Charged pions, kaons, and protons were identified from the energy deposited in the silicon tracker and other track information. In the present analysis, the yield and spectra of identified hadrons for laboratory rapidity $|y | \!<\! 1$ have been studied as a function of the event charged particle multiplicity in the range $|\eta |\!<\!2.4$. The $p_{\mathrm {T}}$ spectra are well described by fits with the Tsallis–Pareto parametrization. The ratios of the yields of oppositely charged particles are close to one, as expected at mid-rapidity for collisions of this energy. The average $p_{\mathrm {T}}$ is found to increase with particle mass and the event multiplicity. These results are valid under the assumption that the particle yield distributions follow the Tsallis–Pareto function in the unmeasured $p_{\mathrm {T}}$ regions.

The results can be used to further constrain models of hadron production and contribute to the understanding of basic non-perturbative dynamics in hadron collisions. The Epos Lhc event generator reproduces several features of the measured distributions, a significant improvement from the previous version, attributed to a new viscous hydrodynamic treatment of the produced particles. Other studied generators (Ampt, Hijing) predict steeper $p_{\mathrm {T}}$ distributions and much smaller $\langle p_{\mathrm {T}} \rangle$ than found in data, as well as substantial deviations in the $\mathrm {p}/{\pi }$ ratios.

Combined with similar results from pp collisions, the track multiplicity dependence of the average transverse momentum and particle ratios indicate that particle production at LHC energies is strongly correlated with event particle multiplicity in both pp and pPb interactions. For low track multiplicity, pPb collisions appear similar to pp collisions. At high multiplicities, the average $p_{\mathrm {T}}$ of particles from pPb collisions with a charged particle multiplicity of $N_\mathrm{tracks}$ (in $|\eta |<2.4$) is similar to that for pp collisions with $0.55 \times N_\mathrm{tracks}$. Both the highest-multiplicity pp and pPb interactions yield higher $\langle p_{\mathrm {T}} \rangle$ than seen in central PbPb collisions.
